# An important role for RPRD1B in the heat shock response

**DOI:** 10.1128/mcb.00173-22

**Published:** 2022-09-19

**Authors:** Simona Cugusi, Prashanth Kumar Bajpe, Richard Mitter, Harshil Patel, Aengus Stewart, Jesper Q. Svejstrup

**Affiliations:** 1 Mechanisms of Transcription Laboratory, The Francis Crick Institute, 1 Midland Road, London, NW11AT, UK; 2 Bioinformatics and Biostatistics, The Francis Crick Institute, 1 Midland Road, London, NW1 1AT, UK; 3 Department of Cellular and Molecular Medicine, Panum Institute, Blegdamsvej 3B, University of Copenhagen, 2200 Copenhagen N, Denmark

## Abstract

During the heat shock response (HSR), heat shock factor (HSF1 in mammals) binds to target gene promoters, resulting in increased expression of heat shock proteins that help maintain protein homeostasis and ensure cell survival. Besides HSF1, only relatively few transcription factors with a specific role in assuring correctly regulated gene expression during the HSR have been described. Here we use proteomic and genomic (CRISPR) screening to identify a role for RPRD1B in the response to heat shock. Indeed, cells depleted for RPRD1B are heat shock sensitive and show decreased expression of key HSPs. These results add to our understanding of the connection between basic gene expression mechanisms and the HSR.

## Introduction

The heat shock response (HSR) is a highly conserved transcriptional program activated in response to a wide range of environmental and intrinsic proteotoxic stresses. The most studied example of such stress is heat shock (HS) (Lindquist, 1986; Morimoto, 1998). Heat shock factor (HSF1 in mammalian cells) is the master regulator of the HSR. Following an insult, HSF1 is activated and binds to target genes containing heat shock promoter elements, inducing their transcription and thus a substantial increase in the synthesis of heat shock proteins (HSPs) (Li et al., 2017). Many HSPs are protein chaperones that act by supporting the folding of newly synthesized proteins, refolding or degradation of misfolded proteins, as well as aggregate solubilization to restore protein homeostasis and ensure cell survival in stress conditions (Hartl et al., 2011).

Alterations of the HSR have been linked to pathological conditions (Gomez-Pastor et al., 2018). For example, the HSR is highly activated in cancer cells, likely enabling them to cope with high levels of proteotoxic stress (Dong et al., 2019; Prince et al., 2020). Moreover, suboptimal activation of the HSR is correlated with neurodegenerative diseases characterized by the presence of protein aggregates such as in Alzheimer’s and Parkinson’s (San Gil et al., 2017). There is therefore a growing interest in understanding the details of the HSR also for therapeutic purposes.

HSF1 interacts with factors that can promote transcription (chromatin remodelers, chromatin modifiers and other transcription factors) suggesting the establishment of a context generally favorable to transcription at HS genes (Gomez-Pastor et al., 2018; Kmiecik and Mayer, 2022; Vihervaara et al., 2018). The HS-induced genes are also the paradigmatic example of so-called “paused genes”: in unstressed conditions, RNA polymerase II (RNAPII) is engaged in their promoter-proximal region, in a paused transcriptional state (Adelman and Lis, 2012). It has been proposed that HSF1 binding facilitates the release of the paused polymerase through the recruitment of CDK9 kinase (Lis et al., 2000; Mahat et al., 2016). The focus of the heat shock field has traditionally been on HSF1 and its partners, while the question of whether RNAPII itself interacts with specific co-factors during HS has not been reported.

RPRD1B (regulation of nuclear pre-mRNA-domain-containing 1B), also known as CREPT (Cell cycle-related and expression-elevated in tumors), contains a C-terminal domain (CTD)-interacting domain (CID), which mediates its interaction with the CTD of the RPB1 subunit of RNAPII (Li et al., 2021). RPRD1B has a paralogue, RPRD1A; the two proteins can dimerize *in vitro* and appear to perform independent as well as overlapping cellular roles. Interestingly, numerous studies have shown that RPRD1B is highly expressed in a majority of solid tumors, where it promotes cell proliferation, tumor growth and progression. Furthermore, its levels correlate with poor prognosis and overall survival (Li et al., 2018; Liu et al., 2019; Lu et al., 2012; Ma et al., 2020; Ma et al., 2017; She et al., 2014; Wang et al., 2014b; Wen et al., 2020; Yin et al., 2019; Zhang et al., 2018; Zheng et al., 2016). RPRD proteins can modulate CTD Serine 5 (Ser5) and Ser7 phosphorylation levels, likely through the recruitment of the phosphatase RPAP2 (Ni et al., 2011; Ni et al., 2014). While the general function of RPRD1B in transcription remains poorly understood, there are indications that it may be involved in multiple steps of the transcription cycle. Like its yeast Rtt103 orthologue, RPRD1B was shown to have a role in transcription termination at a few genes where its depletion resulted in the accumulation of RNAPII downstream of the transcript cleavage site and reduced binding of the termination factor XRN2 (Kim et al., 2004; Morales et al., 2014). RPRD1B is also associated with the promoter of some genes, where it enhances the activity of the TCF4-β-catenin complex to promote transcription (Zhang et al., 2014). Furthermore, it has been proposed that in cancer cells, RPRD1B favors the formation of a loop at the *CCND1* gene to allow the recycling of polymerase from the transcription termination site to the promoter (Lu et al., 2012). Genome-wide analysis of RNAPII CTD modifications in mouse cells with reduced RPRD1B expression indicate that it may participate in the interplay between CTD acetylation and phosphorylation during the initial stages of transcription elongation (Ali et al., 2019). Despite the global changes in the CTD phosphorylation and acetylation status after modest RPRD1B depletion, minimal changes in gene expression were detected in these cells. While RPRD1B may be a general accessory factor in transcription, it is also possible that it becomes especially important in distinctive circumstances, such as during development or in response to stress, such as during the HSR.

In this study, we show that, surprisingly, the transcription of HS induced genes is largely resistant to DRB inhibition of CDK9 activity. We use this as a starting point to identify RPRD1B as an RNAPII-associated factor during HS, which is required for proper expression of HS genes and thus survival upon heat stress. The consequences of these findings for the role of RPRD1B in tumorigenesis are discussed.

## Results And Discussion

### Heat shock induced genes are substantially resistant to DRB inhibition

We previously used DRB/TT_chem_-seq to measure the effect of HS on transcript elongation rates at the genome-wide level (Cugusi et al., 2022). Transient transcriptome sequencing (TT_chem_-seq) (Gregersen et al., 2019; Schwalb et al., 2016) combines a short window of RNA labelling *in vivo* with a chemical RNA fragmentation step prior to deep sequencing, providing a snapshot of the transcription dynamics across all transcribed regions occurring in a specific time frame.

In DRB/TT_chem_-seq (Gregersen et al., 2020) (Figure S1), the CDK9 inhibitor 5,6-dichloro-1-beta-Dribofuranosylbenzimidazole (DRB) is used to restrict RNA polymerase II (RNAPII) to the promoter proximal region. After release from DRB inhibition, transcription progression into the genes is then assessed over time by TT_chem_-seq.

**Figure 1:**
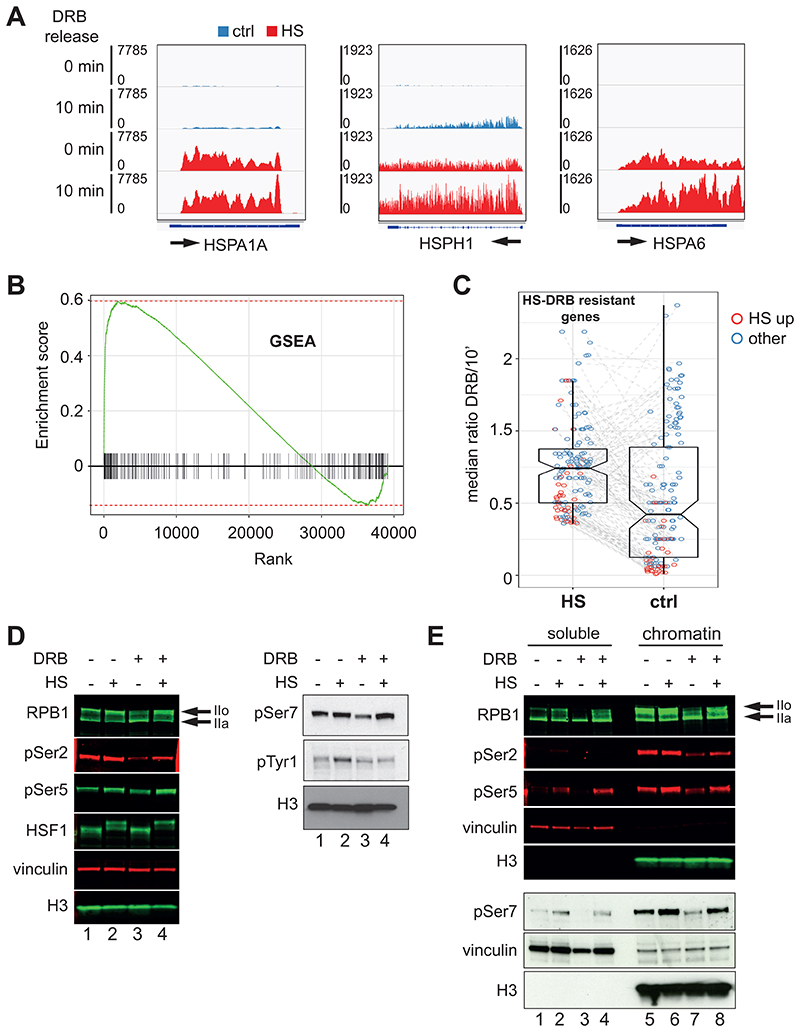
A subset of HS genes are partially DRB-resistant. *(A)* IGV genome browser views of DRB/TT_chem_-seq. Arrows indicate the direction of transcription. (*B*) GSEA analysis showing the degree of overlap between the HS treatment (with genes ranked from most up-regulated (far left) to most down-regulated (far right)) and genes resulting in DRB resistance. The green line represents a running enrichment score. (*C*) relative DRB resistance distribution of the identified HS-DRB resistant genes with (HS) or without HS (ctrl). The colour code represents the status of the genes in response to HS: induced (HS up) or not (other). (*D*) Western blot analysis of RNAPII phosphorylation in whole cell extracts. (*E*) Western blot analysis of RNAPII phosphorylation in chromatin fractionation. Vinculin and H3 are used as loading controls. IIo and IIa indicate respectively the hyperphosphorylated and the hypophosphorylated form of RNAPII. The experiments were carried out in MRC5-VA cells.

During data analysis, we noticed that several classical HS-induced genes were transcribed at similarly high levels before and after release from DRB (Figure 1A); they were DRB-insensitive. This was in striking contrast to most other genes, at which transcription was largely absent in the presence of DRB (see examples in Figure S2A). In order to identify the entire subset of genes evading DRB inhibition in HS, we used the merged data of the two DRB/TT_chem_-seq replicates and calculated the median ratio of reads in the presence of DRB relative to 10 minutes without DRB. This approach returned a list of 267 genes that retained at least 35% of their transcription level even in the presence of DRB (FDR 0.05) (Table S1). We then performed a Gene Set Enrichment Analysis (GSEA) and compared this group of genes to a reference list of genes ranked by their level of differential gene expression in the presence of HS (Figure 1B), according to our previously published nascent RNA data set (Cugusi et al., 2022). The normalized enrichment score (NES) indicated a strong correlation between the DRB resistant genes and the genes most upregulated by HS (NES 2.14, padj <1e-10). Together, these data indicate that genes which are highly activated by HS are insensitive to DRB inhibition. To investigate whether these genes are intrinsically DRB-resistant or become so only after exposure to HS, we performed median ratio analysis (DRB *versus* 10 min DRB release), for the same subset of genes in control (non-HS) samples (Table S1). By plotting the results relative to their differential expression status (HS-induced or not), we detected two clear subgroups: a group of 84 genes that is intrinsically resistant to DRB, mainly represented by genes which are not activated by HS, and a group of 183 genes that are resistant to DRB in a HS-specific manner, largely formed of strongly HS-induced genes (Figure 1C). Analysis of the DRB/TT_chem_-seq data keeping the two replicates separate gave similar results (Figure S2B). We note that most of the genes intrinsically resistant to DRB are histone genes (Table S1 and Figure S2C); this agrees with previous reports showing that CDK9 is not required for histone gene transcription (Medlin et al., 2005; Pirngruber et al., 2009). Some well-known HS genes, such as DNAJB1 and HSP90AA1 do not figure in the list in Table S1. However, manual inspection of these genes shows that they are also DRB-resistant (Figure S3A). The usage of an alternative TSS located several kbs downstream the TSS of the longest annotated isoform, which is the one considered in the bioinformatics analysis, probably compromise the ability to pass the established thresholds. This suggests that DRB-resistance is likely to be a general feature of HS-genes but to obtain a comprehensive list of the genes that are DRB-resistant would require manual inspection of the entire genome. DRB-resistant transcription was confirmed by nascent RNA qPCR at a couple of HS-induced genes (Figure S3B, left panel).

Surprisingly, DRB-resistant transcription did not result in corresponding production of stable mRNA (Figure S3B, right panel), indicating that DRB affects the correct processing of the affected transcripts and thus that CDK9 also plays a role beyond transcription pause-release, as previously suggested by others (Laitem et al., 2015; Ni et al., 2004). In our experiments, treatment with DRB preceded HS, and inhibition appears to be equally effective in control and HS conditions at non-induced genes (Figure S2A), indicating that the induction of HS genes overcomes a previously established inhibition of transcription. We were surprised by these findings since HS genes represent the paradigmatic example of regulation through promoter-proximal pausing and CDK9 is known to be recruited to HS genes and stimulate their transcription (Boehm et al., 2003; Lis et al., 2000).

Because RPB1 CTD phosphorylation is a marker of actively transcribing RNAPII and is abrogated by treatment with DRB (Dubois et al., 1994; Lavoie et al., 2001; Medlin et al., 2003), we checked CTD phosphorylation status. In agreement with other reports (Dubois et al., 1994; Dubois et al., 1997), we found that RNAPII becomes more hyperphosphorylated (II0) during HS (Figure 1D and 1E, compare lane 1 and 2 and 5 and 6). This increase in phosphorylation is mainly due to an increase in pSer5, pSer7 and pTyr1, while pSer2 appears to be relatively unchanged. Notably, after DRB treatment alone, the levels of phosphorylated RNAPII were substantially reduced, while they were increased again in the samples subjected to both DRB treatment and HS (Figure 1D and 1E; compare lanes 1, 3 and 4 and 5, 7 and 8). More specifically, pSer5, pSer7 and pSer2, were all clearly increased after HS in DRB-inhibited conditions. DRB-resistant phosphorylation of RNAPII was also observed in other two cell lines, HEK293 and U2OS (Figure S3C).

Collectively, these results indicate that HS can overcome CDK9 inhibition of transcription at many induced genes and that the relief of inhibition can also be observed at the level of RNAPII CTD phosphorylation.

### Identification of new factors involved in the HS response

**Figure 2:**
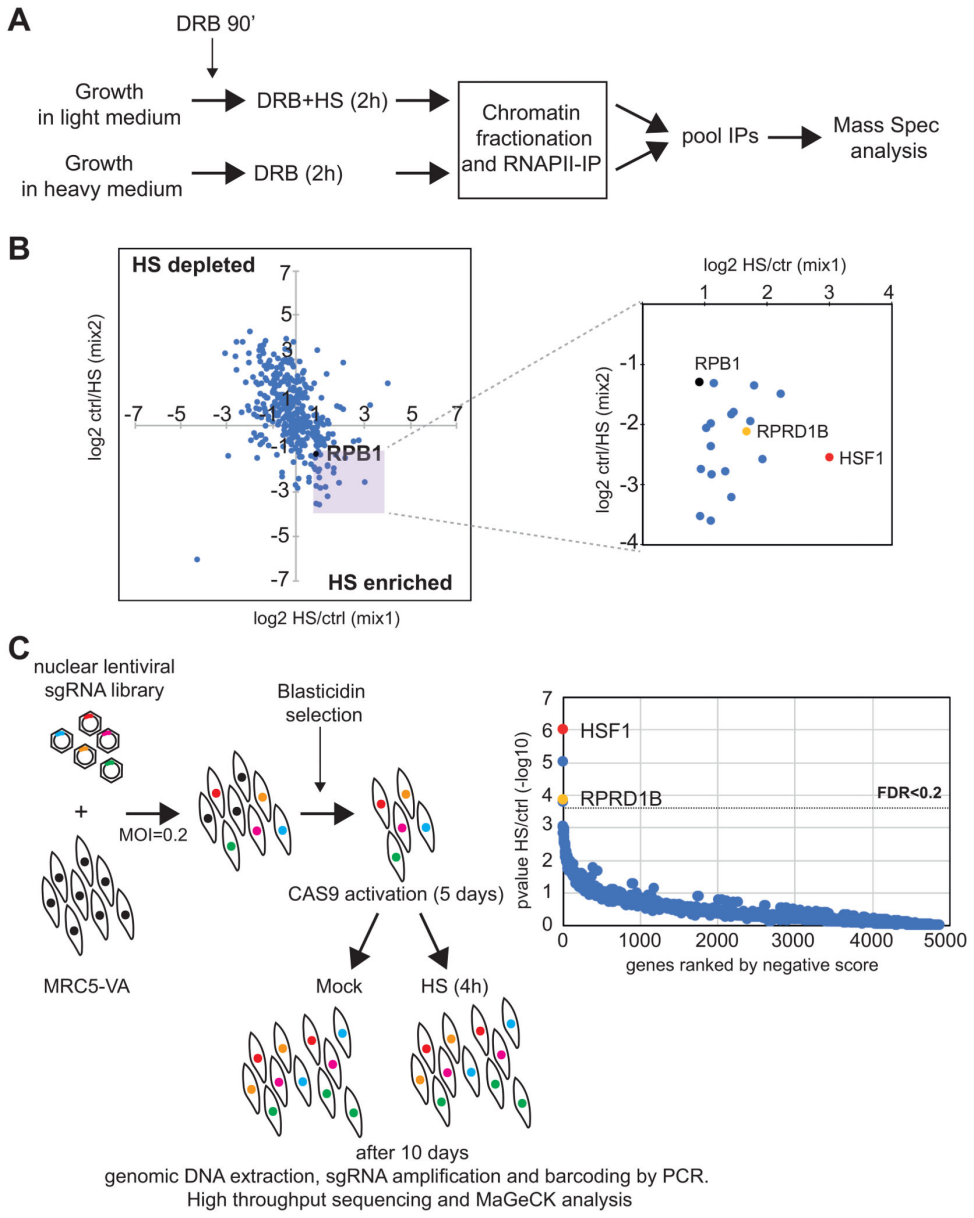
Identification of new factors involved in HSR. *(A)* Schematic representation of IP-mass spec experiment. (*B*, *left*) IP-mass spec results plot. (Right) Interactors enriched after HS with respect to RPB1 amount. (*C*, *left*) CRISPR screen scheme. (*Right*) Average gene depletion results plot. The experiments were carried out in MRC5-VA cells.

While we have so far failed to uncover the mechanistic basis for HS-mediated escape from DRB inhibition of transcription and CTD phosphorylation, we considered that the findings might be exploited to identify new factors specifically involved in transcription of HS genes. Indeed, in cells treated with DRB and then subjected to heat shock, RNAPII activity at strongly HS-induced genes represent the main category of RNAPII transcription observed across the genome. We thus performed immuno-precipitation of RNAPII coupled with mass spectrometry (IP-MS) using quantitative stable isotope labelling by amino acids in cell culture (SILAC) (Figure 2A and Table S2). Briefly, RNAPII was immunoprecipitated from chromatin fractions of DRB-treated cells exposed to HS or not (Figure S4A). HS and control IP eluates were pooled and analyzed by mass spectrometry.

As expected, RPB1 was highly abundant in both control and HS samples (Figure S4B). The two mixes correlated well (Figure 2B, left), with RPB1 appearing to be slightly enriched in the HS samples. This might be explained by the preference of the 4H8 antibody for the phosphorylated form of RPB1. We therefore restricted our analysis to the proteins that were enriched relative to RPB1 (Figure 2B, right). Among those, we identified proteins with a known function in HS, such as the main transcriptional activator, HSF1, and Mediator subunits, which play an important role in the activation of the HS genes (Kim and Gross, 2013; Park et al., 2001). Intriguingly, one of the best scoring hits was RPRD1B, a known CTD-binding protein whose function in transcription is still poorly understood, but which has not previously been linked to the HS response.

In a parallel effort to identify new factors relevant for the HS response, we performed a CRISPR-Cas9 depletion screen (Figure 2C). Briefly, we generated a MRC5-VA cell line expressing Dox-inducible Cas9. These cells were then transduced with a library of lentiviral single guide RNAs (sgRNAs) targeting nuclear factors (Wang et al., 2014a). Five days after Cas9 activation to initiate gene disruption, the cells were exposed to HS for four hours. After ten days, the resulting cells were harvested, and genomic DNA extracted for PCR amplification. sgRNA abundance was quantified by next-generation sequencing and compared to untreated cells using model-based analysis of genome-wide CRISPR-Cas9 knockout (MAGeCK; (Li et al., 2014)) (Figure 2C and Table S3). Gratifyingly, HSF1 scored as the top hit, validating the screen. Otherwise, we identified only a few significant hits (FDR <0.2) but RPRD1B was one of them (Figure 2C, yellow sphere). An evaluation of HSF1 and RPRD1B single sgRNAs performance in the screen showed that the level of depletion of RPRD1B sgRNAs was comparable to the majority of the sgRNAs targeting HSF1 (Figure S4C). Intrigued by the recurrence of RPRD1B using fundamentally different approaches, we decided to further investigate its function in the HS response.

### RPRD1B is required for optimal induction of HSPs

**Figure 3:**
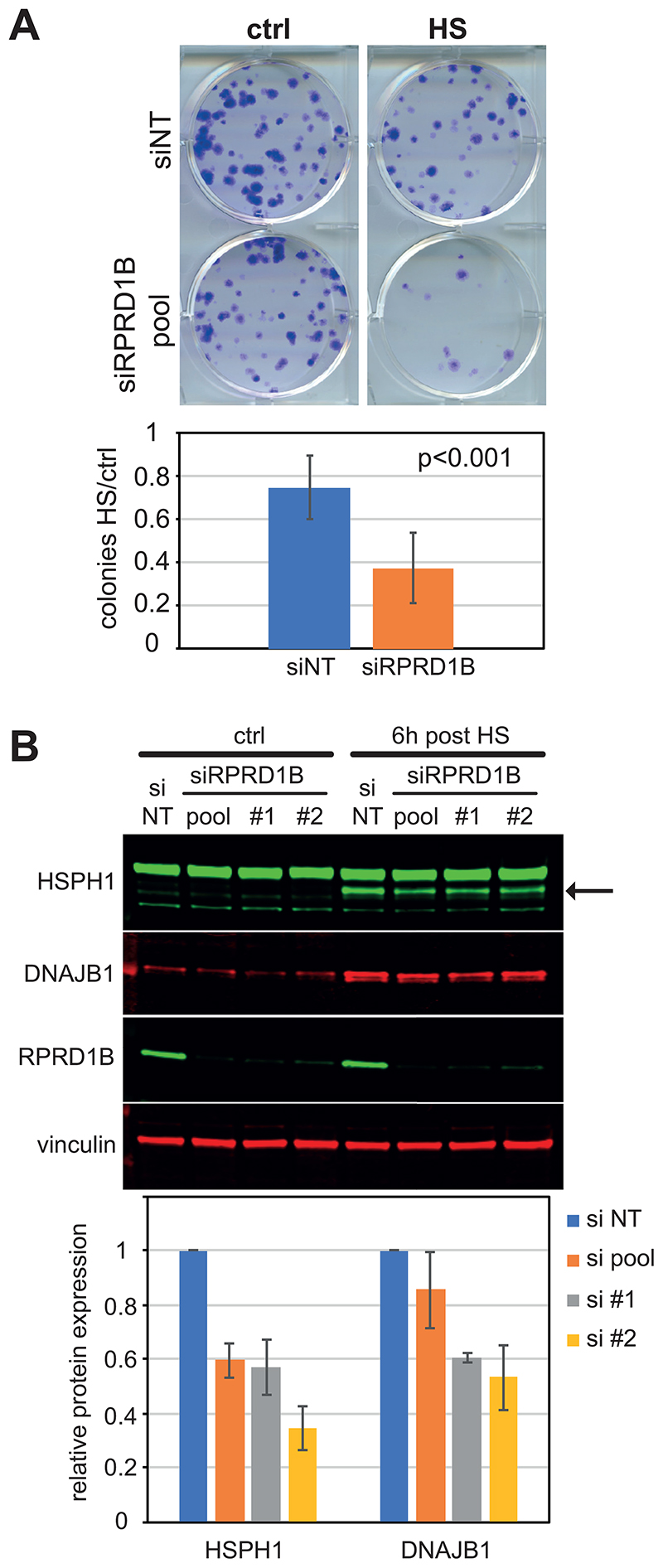
RPRD1B is required for optimal HSR. (*A, top*) Representative example of colony formation for HS survival assay. (*Bottom*) Colony counting from HS treated cells normalised to untreated ctrl. Average of four biological replicates. Error bars indicate ±SD. (*B*, *top*) Western blot analysis of HSPs induction in whole cell extracts of cells . Vinculin is loading control. Arrow indicates the HS-induced isoform of HSPH1. (*Bottom*). Relative quantification of fluorescence intensity. Average of three biological replicates. Error bars indicate ±SD. The experiments were carried out in MRC5-VA cells and siRPRD1B was depleted either with a mix of four siRNAs (pool) or two single siRNAs (#1 and #2)

To confirm that RPRD1B is important for HS survival, we performed a HS survival assay by colony formation in cells depleted for RPRD1B by a pool of siRNAs (Figure 3A, upper panel). The siRNA treatment efficiently knocked down RPRD1B but not its paralogue RPRD1A (Figure S5A). An average of four biological replicates showed that RPRD1B depletion results in a significant reduction in HS survival (Figure 3A, lower panel).

We then asked if this heat sensitivity could be explained by a defect in the induction of heat shock proteins (HSPs) by testing the protein-level expression of two well-known HSPs, HSPH1 and DNAJB1, in cells depleted for RPRD1B. We first performed a time-course experiment to identify the optimal time point to detect the induction of HSPs (Figure S5B). The results led us to perform the comparison six hours after HS. Interestingly, RPRD1B depletion, either by a pool of four siRNAs or by two different single siRNAs, correlated with up to 65% reduction of the heat-induced isoform of HSPH1 and up to 45% reduction in DNAJB1 level (Figure 3C). By contrast, the expression of these proteins does not seem to require RPRD1B in the absence of HS (Figure S5C). HSF1 appeared to be activated normally in the absence of RPRD1B, as indicated by its phosphorylation status (Figure S6A), as well as by the formation of the characteristic nuclear foci (Figure S6B). We then explored whether RPRD1B contributes to evade DRB transcription inhibition during HS. For this purpose, after RPRD1B knock down, the expression of nascent HSPH1 was measured by qPCR analysis in the presence or absence of DRB treatment, in cells grown at standard temperature (NHS) or in cells exposed to HS (Figure S6C and S6D). The results show that cells depleted for RPRD1B show comparable levels of DRB-resistance respect to the control cells.

Collectively, these results confirm that RPRD1B is required for survival after HS and indicate that key HSPs depend on RPRD1B for their optimal HS-induced expression.

### RPRD1B promotes the transcription of HS genes

**Figure 4:**
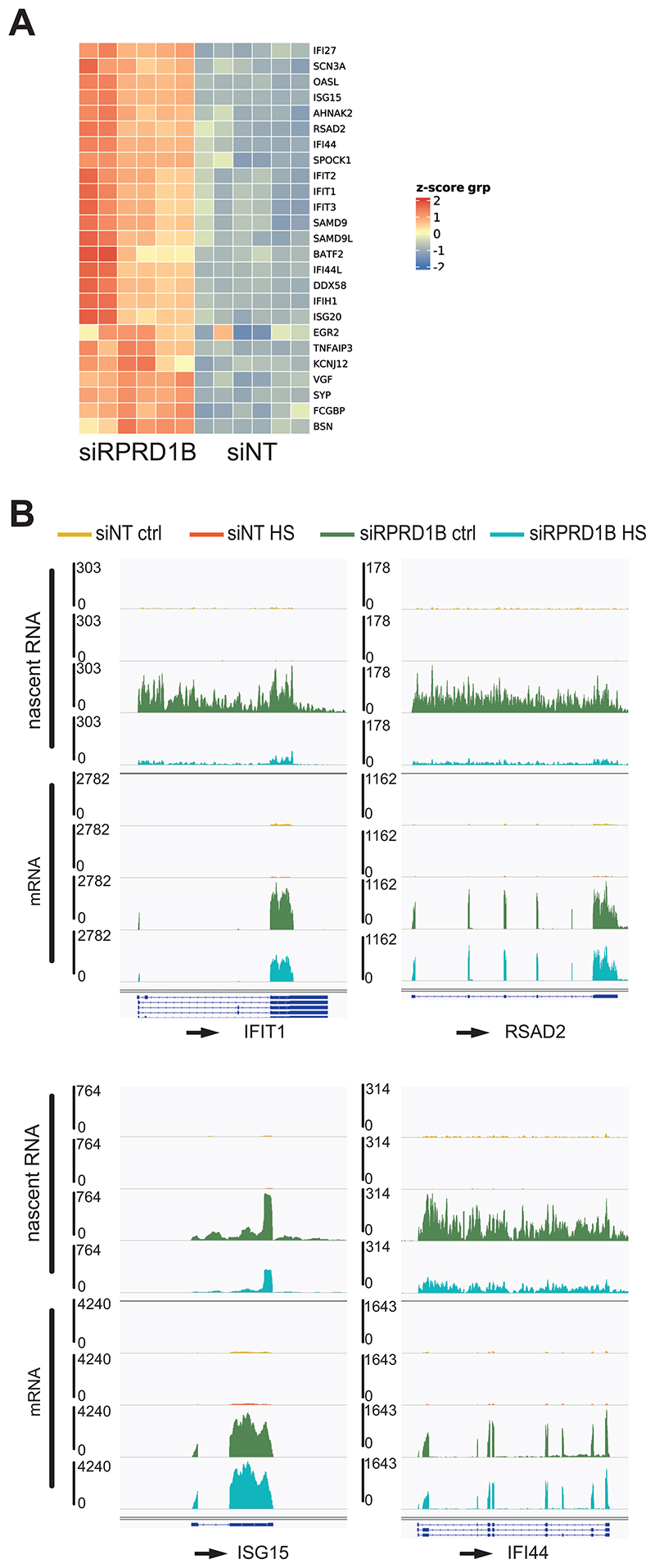
RPRD1B depletion results in ISGs induction. *(A)* Heatmap representing the top upregulated genes after RPRD1B depletion. (*B*) IGV genome browser views of TT_chem_-seq of the indicated genes. The experiments were carried out in MRC5-VA cells.

The RNAPII mass spec results opened the possibility that RPRD1B interacts with RNAPII at HS-induced genes to promote their expression. Generally, limited information exists on the set of genes affected by RPRD1B depletion. In order to obtain a genome-wide view of nascent RNA- and mRNA production, we performed mRNA-seq and TT_chem_-seq after HS treatment in cells depleted for RPRD1B (Figure S7A). We first performed differential gene expression analysis, comparing control cells and cells knocked down for RPRD1B, in the presence or absence of HS, using the mRNA-seq data set (Table S4). Approximately 400 genes were upregulated by RPRD1B knockdown in both conditions (2-fold change; FDR 0.05). Intriguingly, interferon-stimulated genes (ISGs) were among the most upregulated genes (Figure 4A). Single gene examples confirmed this finding, not only at the mRNA level but also at the level of nascent transcription (Figure 4B). Interestingly, HS appeared to repress, and in some instances completely abolish, the ISG induction observed in RPRD1B-depleted cells, as is evident from the nascent RNA profile (TT-Seq). At some genes, HS-mediated repression was also detectable at the mRNA level, despite the relatively short HS treatment. These results suggest that RPRD1B depletion activates ISG expression, and that HS suppresses such activation.

Metagene analysis of nascent RNA showed clear evidence for the characteristic global downregulation of genome-wide transcription in response to HS, with a similar behavior for RPRD1B knock-down and control cells (Figure S7B). Most importantly, however, we investigated whether RPRD1B depletion results in suboptimal induction of HS-induced genes.

**Figure 5:**
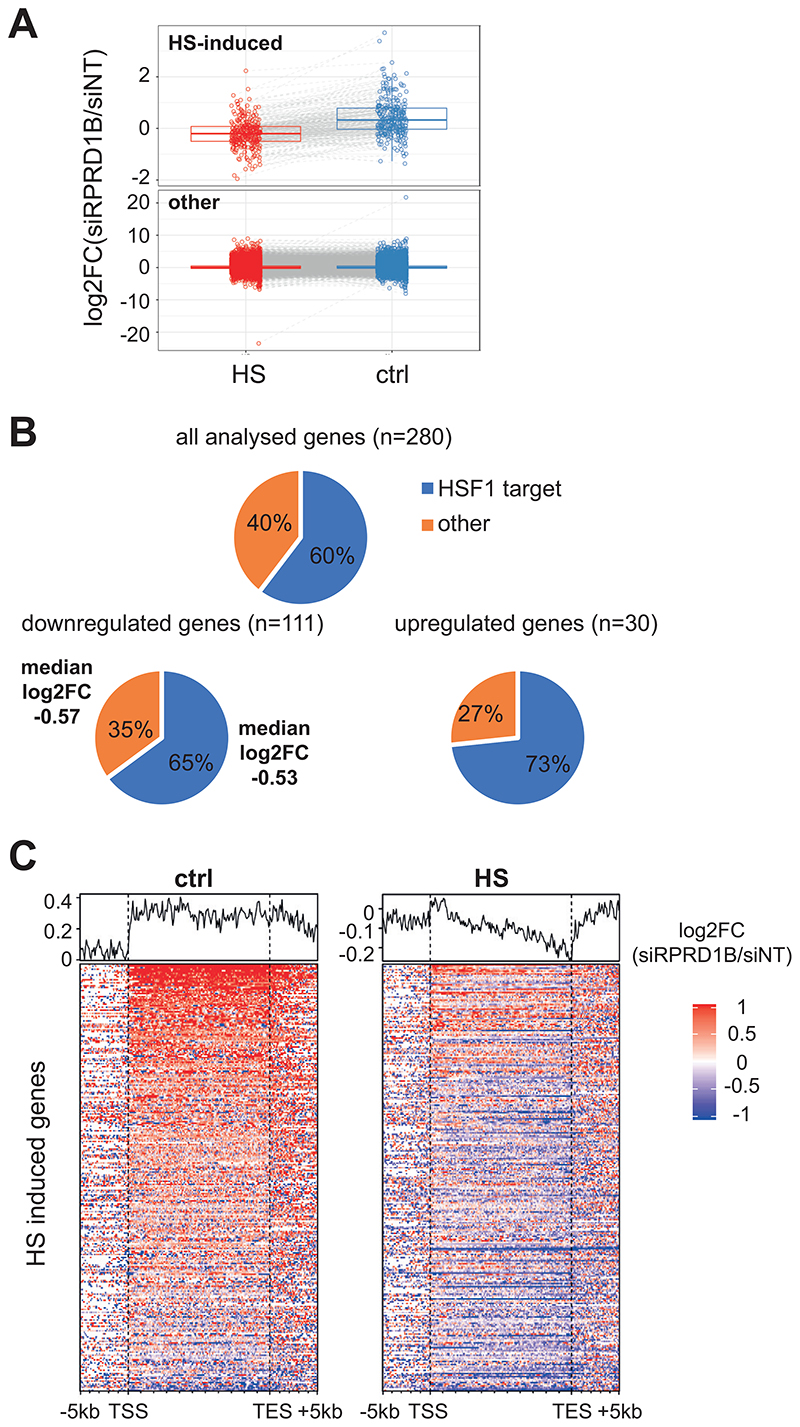
RPRD1B sustains HS genes induction. *(A)* Relative mRNA expression of HS-induced and non-HS-induced (other) genes after RPRD1B depletion. (*B*, *top*) proportion of known targets of HSF1 (HSF1 target) and genes not regulated by HSF1 (other) among the HS induced genes. (*B*, *bottom*) proportion of known targets of HSF1 (HSF1 target) and genes not regulated by HSF1 (other) among the HS induced genes showing significant differential gene expression (siRPRD1B HS *vs* siNT HS). (*C*) Heatmaps of nascent RNA relative abundance at HS-induced genes. The profiles on top represent an average of the analysed genes. The experiments were carried out in MRC5-VA cells.

To first identify and characterize the HS-induced genes in our dataset, we performed differential gene expression analysis on the mRNA-seq data in control cells exposed to HS, which returned 280 up-regulated genes (2-fold change; FDR 0.05) (Table S5). When focusing on the overall expression of this HS-induced group of genes at the mRNA level, we found that these genes are less expressed after HS in cells depleted for RPRD1B, while this was by and large not the case at normal temperature (Log2FC median difference -0.555) (Figure 5A, upper). By contrast, similar analysis of the rest of the genes (‘other’, lower) showed no significant difference between HS and control conditions (Log2FC median difference 0.001), confirming that RPRD1B depletion does not result in a general downregulation of gene expression, but that it preferentially affects HS-induced genes after HS (Wilcoxon test p<0.0001). We then sought to investigate whether RPRD1B’s function in HS is specific to HSF1 target genes or whether it is active on genes induced by alternative pathways. Using HSF1Base database (Kovacs et al., 2019), we found that 169 (approximately 60%) of the HS-induced genes in our cells are known targets of HSF1 (Figure 5B and Table S5). According to the differential expression analysis (siRPRD1B HS *vs* siNT HS in Table S4), of the 280 HS-induced genes, 111 show some level of downregulation (FDR 0.05) and 30 are upregulated (FDR 0.05). Among the downregulated, 72 (65%) are known HSF1targets (Figure 5B). The proportion of HSF1 target genes among the genes downregulated by RPRD1B depletion reflects the proportion of these genes in the population analyzed (n=280). The median difference in expression in the two groups (HSF1 targets and others) is very similar respectively -0.53 and -0.57. These results support the idea that RPRD1B affects HSF1-dependent and -independent HS-induced genes alike.

**Figure 6:**
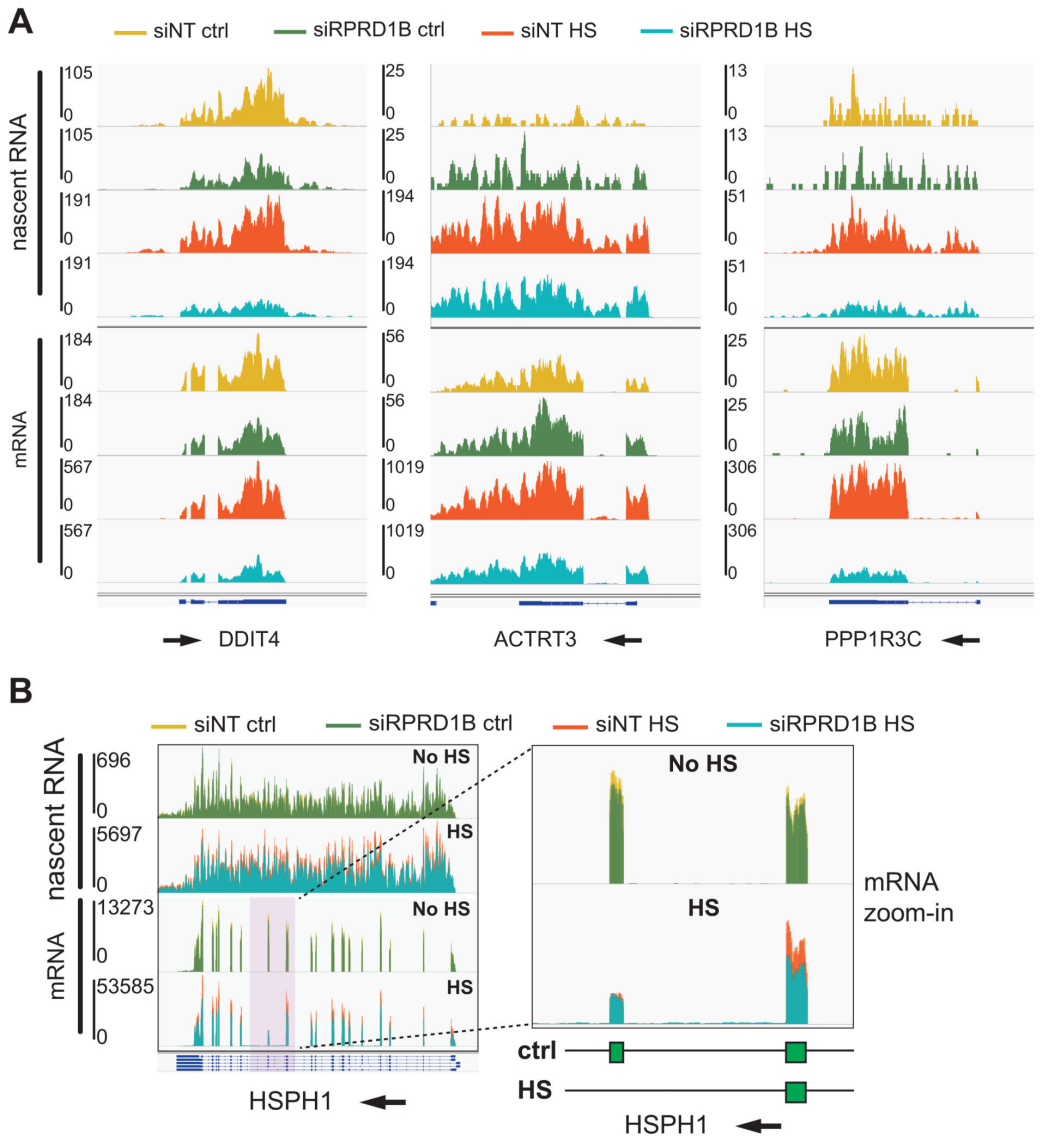
HS induced genes affected by RPRD1B depletion. (*A*) IGV genome browser views of TT_chem_-seq and mRNA-seq. (*B*, *left*) IGV genome browser views of TT_chem_-seq and mRNA-seq of HSPH1 gene. (*Right*) zoom-in detail of *(C)*, exons 11 and 12 of the constitutive isoform of HSPH1 gene are indicated by the green boxes. The experiments were carried out in MRC5-VA cells.

At the level of nascent transcription, the 280 genes show a similarly varied response to the absence of RPRD1B: there is little or no effect in the control conditions (Figure 5C, left), but after HS, a subtle but easily detectable downregulation across the gene body that becomes more pronounced towards the gene 3’-end can be observed (clearly seen in the average trend-curve above the heatmap), suggesting a defect in aspects of transcript elongation (Figure 5C, right). So, RPRD1B appears to be largely dispensable for the basal expression of these genes, but it is required for full induction after HS. Single gene profiles of some of the most downregulated genes, at the mRNA level, in this group exemplify this trend (Figure 6A and S8).

The HS-specific effect is particularly interesting for *HSPH1*. This gene has a constitutive form and a HS-induced mRNA form, which is a product of alternative splicing, resulting in exon skipping. The mRNA-seq data showed that the constitutive form is equally expressed in control and RPRD1B knock down cells, as illustrated by the reads mapping to the exon present only in the constitutive form, while the induced form is clearly downregulated in cells depleted for RPRD1B (Figure 6B, panel on the right).

We also measured the readthrough level beyond the transcription termination site since Rtt103, a putative yeast orthologue of RPRD1B, has a function in transcription termination (Kim et al., 2004) and because such a function was also reported for RPRD1B (Morales et al., 2014). However, we only detected negligible differences in readthrough between cells depleted for RPRD1B and control cells (Figure S9A). mRNA splicing analysis also only showed minimal changes in RPRD1B-depleted cells (Figure S9B); and at genes which are not enriched in the HS-induced genes (Table S6). Together, these results indicate that HS-induced genes require RPRD1B to achieve optimal HS protein levels after HS, and that this occurs through deficiencies in gene expression.

The HSR is the primary cellular defense against proteotoxic stress and works primarily through the induction of a small subset of genes encoding HSPs (Alagar Boopathy et al., 2022; Hipp et al., 2019). Some aspects of HS gene expression are known in great details; however, how the high level of gene expression required is achieved is not completely understood. The growing interest in the role played by the HSR in pathological contexts (Gomez-Pastor et al., 2018), such as in cancer and in neurological diseases further highlights the importance of gaining a deeper insight into the factors and mechanism underlining the HSR.

In this work, we show that HS-induced genes escape DRB inhibition of CDK9 in the general context of the transcriptional repression observed after DRB treatment. We used this as a basis to uncover a new factor involved in the HSR, RPRD1B, which has limited effect on transcription under normal conditions but is required for optimal expression of HS-induced genes and cell survival after HS.

CDK9 is known to be recruited to HS genes following HSF activation and it is even capable of stimulating HS genes in the absence of HSF if artificially recruited at their promoter (Boehm et al., 2003; Lis et al., 2000). Curiously, although CDK9 is thought to play a primary role in HS-induced gene expression (Guertin et al., 2010), genome-wide analysis of HS transcription after CDK9 inhibition uncovered a large group of classical HS genes that are unaffected by DRB treatment. Interestingly, early studies performed with the CDK9 inhibitor DRB in *Drosophila hydei*, *Chironomus tentans* and *Drosophila melanogaster*, either on polytene chromosomes or at single gene loci, actually already suggested the existence of DRB-resistant transcription at HS-induced genes (Egyhazi et al., 1998; Giardina and Lis, 1993; Wiltenburg and Lubsen, 1976). Later on, the usage of another CDK inhibitor, flavopiridol, lead to apparently different outcomes depending on the length of the treatment (Ni et al., 2008; Ni et al., 2004). Our genome-wide results showed that while non-induced genes are repressed by inhibition of CDK9 with DRB, such treatment is of little consequence at most classical HS-induced genes, as well as at the canonical histone genes, whose transcription does not require RNAPII Ser2 phosphorylation (Medlin et al., 2005). The observed DRB-resistant transcription correlates with recovery of RNAPII phosphorylation after HS, implying the involvement of other kinases or the establishment of a new balance between kinases and phosphatases. Interestingly, despite the high level of nascent transcription, a corresponding increase in mRNA levels for HS genes was not observed. This interesting observation appears to be in line with previous reports showing that CDK9 has a checkpoint function in transcription near transcriptional termination sites, and that it is required for proper processing of HS gene transcripts and optimal mRNA production (Laitem et al., 2015; Ni et al., 2004).

While the mechanism underlying DRB-resistant transcription of HS induced genes remains unclear, such transcription proved a useful tool to identify factors that interact with RNAPII specifically while it is engaged at HS-induced genes. By exploiting that DRB-resistant transcription occurs almost exclusively at HS induced genes, we thus identified the RNAPII CTD-binding protein RPRD1B, whose role in the HSR was further supported by the results of a CRISPR screen for HS survival. RPRD1B is required for survival to HS, and for proper expression of HSPs also at the protein level. The fact that RPRD1B interacts with RNAPII while it is mainly transcribing HS-induced genes suggests a direct role in the expression of these genes.

Intriguingly, following RPRD1B depletion, HS-induced genes are most clearly downregulated at the mRNA level. Somewhat in contrast, analysis of nascent RNA production at these genes shows a more subtle effect, with downregulation more obvious in the gene body towards the 3’ end, suggesting a defect in aspects of transcription elongation. In any case, RPRD1B is not generally required for expression of these genes, but primarily for their optimal induction after HS. We note that RPRD1B function in the HS response is unlikely to be due to a direct interaction with HSF1. Indeed, several proteomic studies have established the HSF1 interactome, and RPRD1B was not identified as a partner in these experiments (Burchfiel et al., 2021; Smith et al., 2022; Takii et al., 2019; Zhang et al., 2019). Furthermore, HSF1 appears to be normally activated in cells depleted for RPRD1B. However, it is worth noting that although HSF1 does not seem to require other factors for DNA binding (Gomez-Pastor et al., 2018), we cannot completely rule out the possibility that promoter-specific binding is affected in the absence of RPRD1B at certain genes.

It has previously been reported that RPRD1B depletion results in a genome-wide increase of RNAPII Ser5 phosphorylation and K7 acetylation (Ali et al., 2019). This did not have a clear effect on transcription. However, it is possible that specific local variations of RNAPII modifications might contribute to HS-genes downregulation; this remains to be investigated. We found no dramatic, specific effect on phosphorylation after HS in RPRD1B depleted cells (Figure S10) and note that the observed defect in nascent transcription alone seems unlikely to explain the extent of downregulation at the level of mRNA and protein. It is thus possible that RPRD1B is important not only for the transcription of HS genes but also somehow for the correct processing of their mRNA. Whether RPRD1B is required for proper function of the CDK9-maintained elongation checkpoint near the end of genes (Laitem et al., 2015; Ni et al., 2004) is an interesting possibility that also remains to be investigated.

Finally, RPRD1B is highly expressed in a wide range of cancers and its levels correlate with poor prognosis and overall survival (Li et al., 2021; Lu et al., 2012; She et al., 2014; Yu et al., 2019; Zheng et al., 2016). Moreover, the HSR is appropriated by cancer cells, likely to deal with their high levels of proteotoxic stress. Our findings thus raise the intriguing possibility that RPRD1B’s key role in the HSR might help explain its tumor-promoting function.

## Materials and Methods

### Genome-wide analysis

TTchem-seq was performed as in (Cugusi et al., 2022). mRNAseq was performed as in (Tufegdzic Vidakovic et al., 2020). The supporting datasets were deposited to the NCBI’s Gene Expression Omnibus under accession number GSE197995.

### SILACIP-mass spec

Protein labelling in SILAC medium was performed according to standard procedures. Chromatin fractionation and RPB1 IP were performed as in (Tufegdzic Vidakovic et al., 2020).

### CRJSPR screen

Cas9-MRC5VA cell lines were created by transduction of dox inducible flag-Cas9 vector (pLX-sgAAVS1, Addgene). The Human CRISPR nuclear sub pool library was purchased from Addgene (Wang et al., 2014a), pLX-sg-non targeting-A bunch of 30 non targeting plasmids was a kind gift from provided by Paola Scaffidi. Lentivirus of the nuclear sub pool was generated by co-transfection of the nuclear library sub pool with VSVG, GAG-POL and REV packaging plasmids.

### Western blot

Western blot analysis was performed according to standard procedures. Primary antibodies used: HSF1 (Enzo Life Sciences, ADI-SPA-901); Vinculin (Sigma-Aldrich, V9131); RPRD1B (Bethyl, A303-782A-M); RPRD1A (Proteintech, 23652-1); Histone H3 (Abcam, ab1791); RPB1 N-terminal (Cell Signaling, D8L4Y); RPB1 Serine 2 phosphorylated, Serine 5 phosphorylated, Serine 7 phosphorylated and Tyrosine 1 phosphorylated are a kind gift from Dirk Eick; HSP70 (Proteintech, 25405-1); DNAJB1 (Proteintech, 67422-1); BAG3 (Proteintech, 10599-1); HSPH1 (Proteintech, 13383-1).

For further details, please see the Supplemental Material.

## Supplementary Material

Figure S1

Table S1

Table S2

Table S3

Table S4

Table S5

Table S6

Table S7
