# Supplementary material for: An important role for RPRD1B in the heat shock response
Source: Mol Cell Biol. Author manuscript; Available in PMC 2022 Oct 24. (PMC9583720; doi:10.1128/mcb.00173-22)
Supplement: Figure S1 [file EMS155264-supplement-Figure_S1.pdf]

Supplementary Figures with Legends

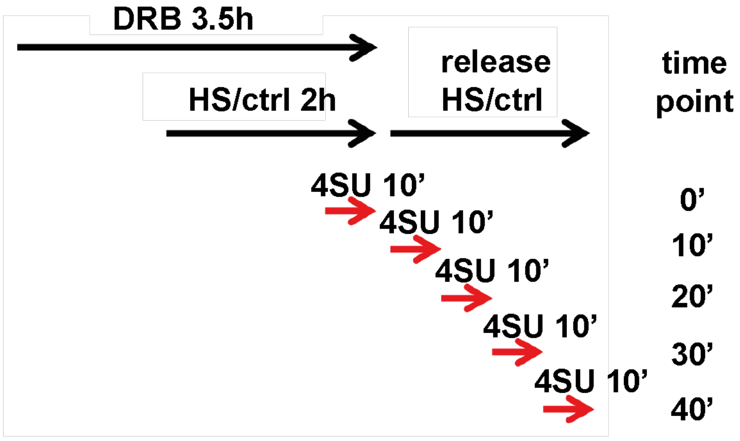

Figure S1. Schematic representation of DRB/TT_{chem}-seq experiment.

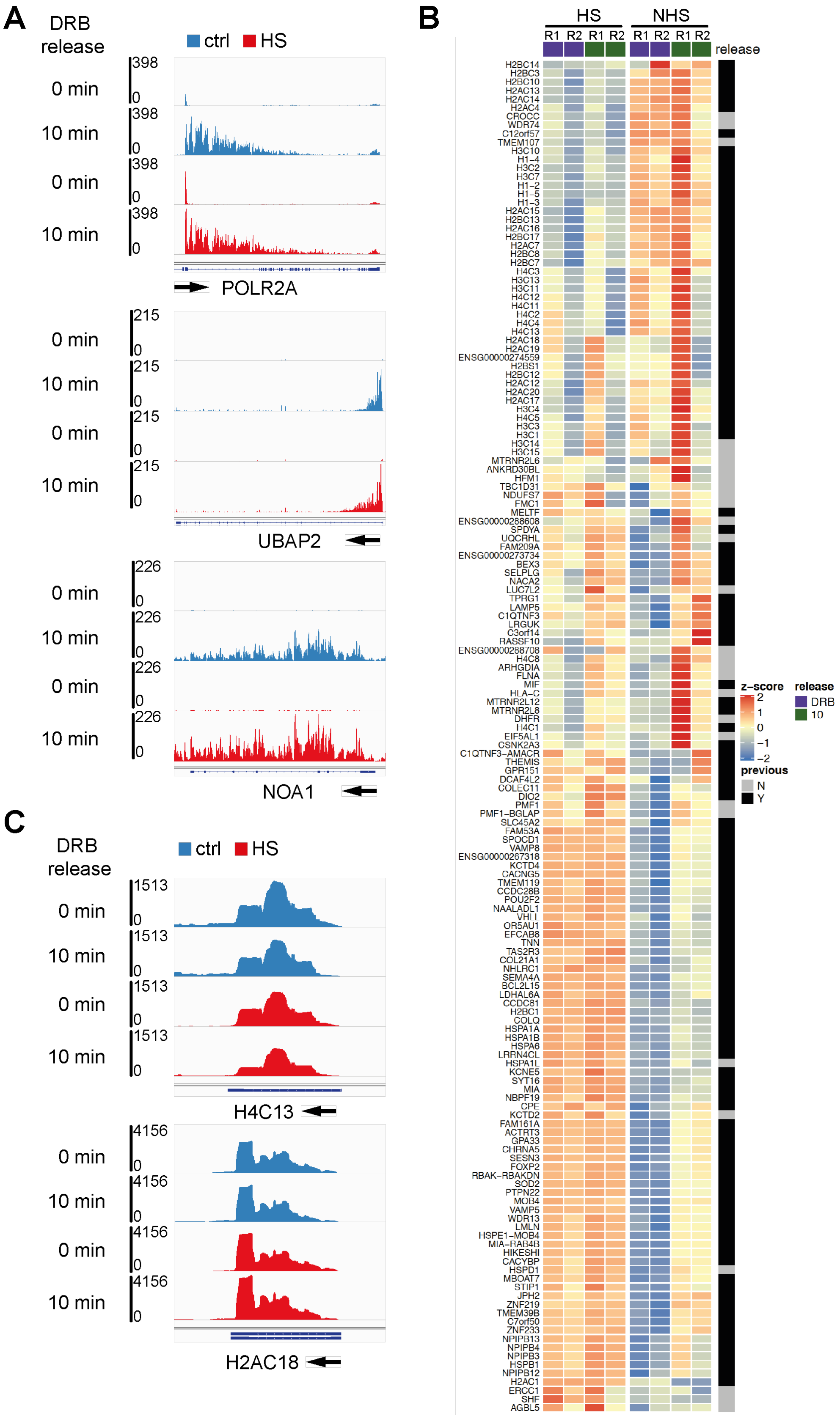

Figure S2. *(A)*IGV genome browser views of DRB/TT_{chem}-seq of the indicated genes. *(B)* Heatmap of HS-DRB resistant genes obtained by analysing the replicates separately. Previous refers to the presence in the list of DRB resistant genes in Table S1. *(C)* IGV genome browser views of DRB/TT_{chem}-seq of the indicated genes. The experiments were carried out in MRC5-VA cells.

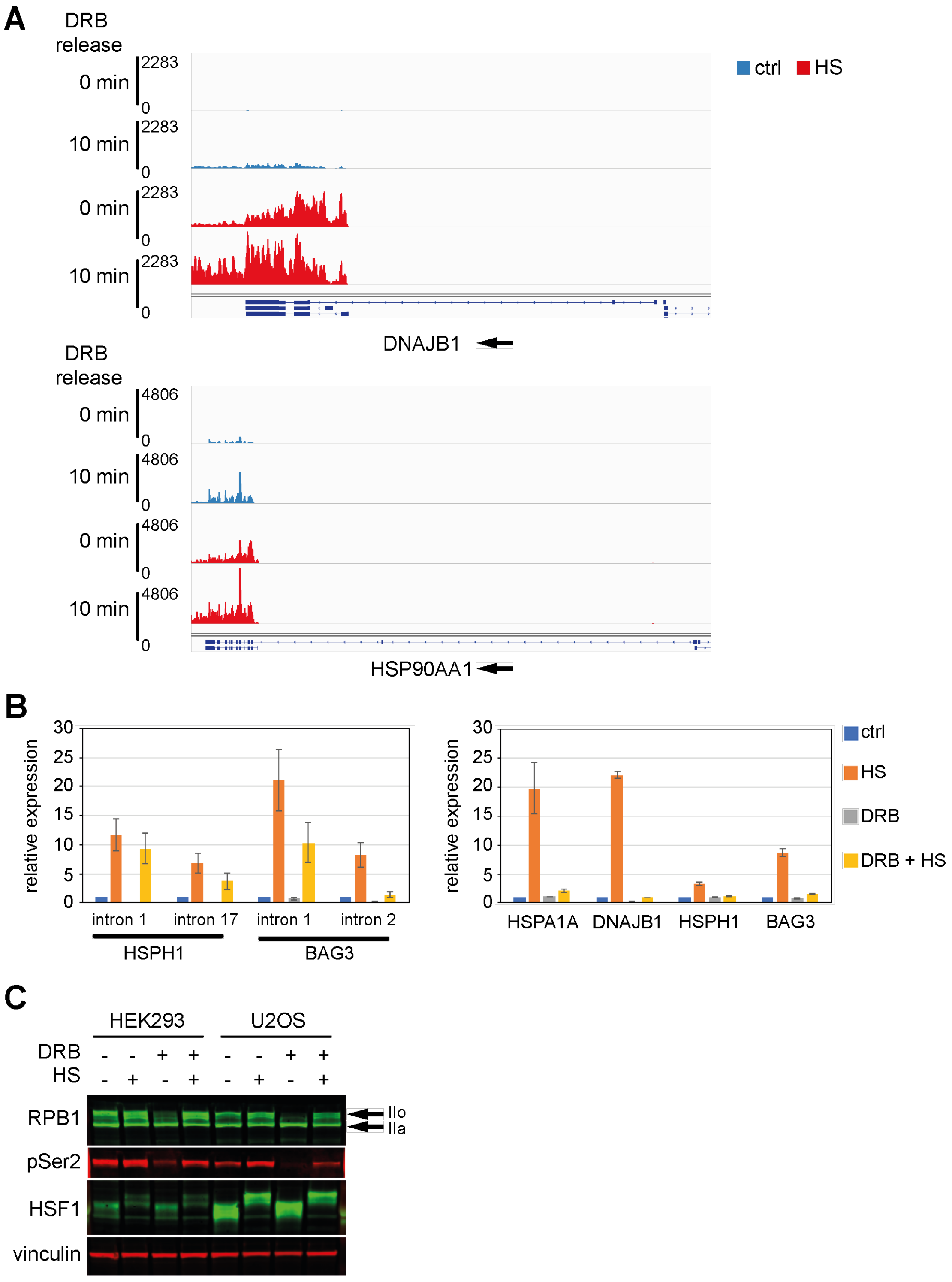

Figure S3. *(A)*IGV genome browser views of DRB/TT$_{chem}$-seq of the indicated genes. The experiments were carried out in MRC5-VA cells. (*B,left*) qPCR quantification of nascent RNA near the 5'-end or the 3'-end of the indicated genes, relative to *GAPDH*, normalized to the control. *(Right)* qPCR quantification of mRNA of the indicated genes, relative to *GAPDH*, normalized to the control. Average of three biological replicates; error bars indicate ±SD. The experiments were carried out in MRC5-VA cells. (*C*) Western blot analysis of RNAPII phosphorylation in whole cell extracts. Vinculin is loading control. IIo and IIa indicate respectively the hyperphosphorylated and the hypophosphorylated form of RNAPII. The experiments were carried out in U2OS and HEK293 cells.

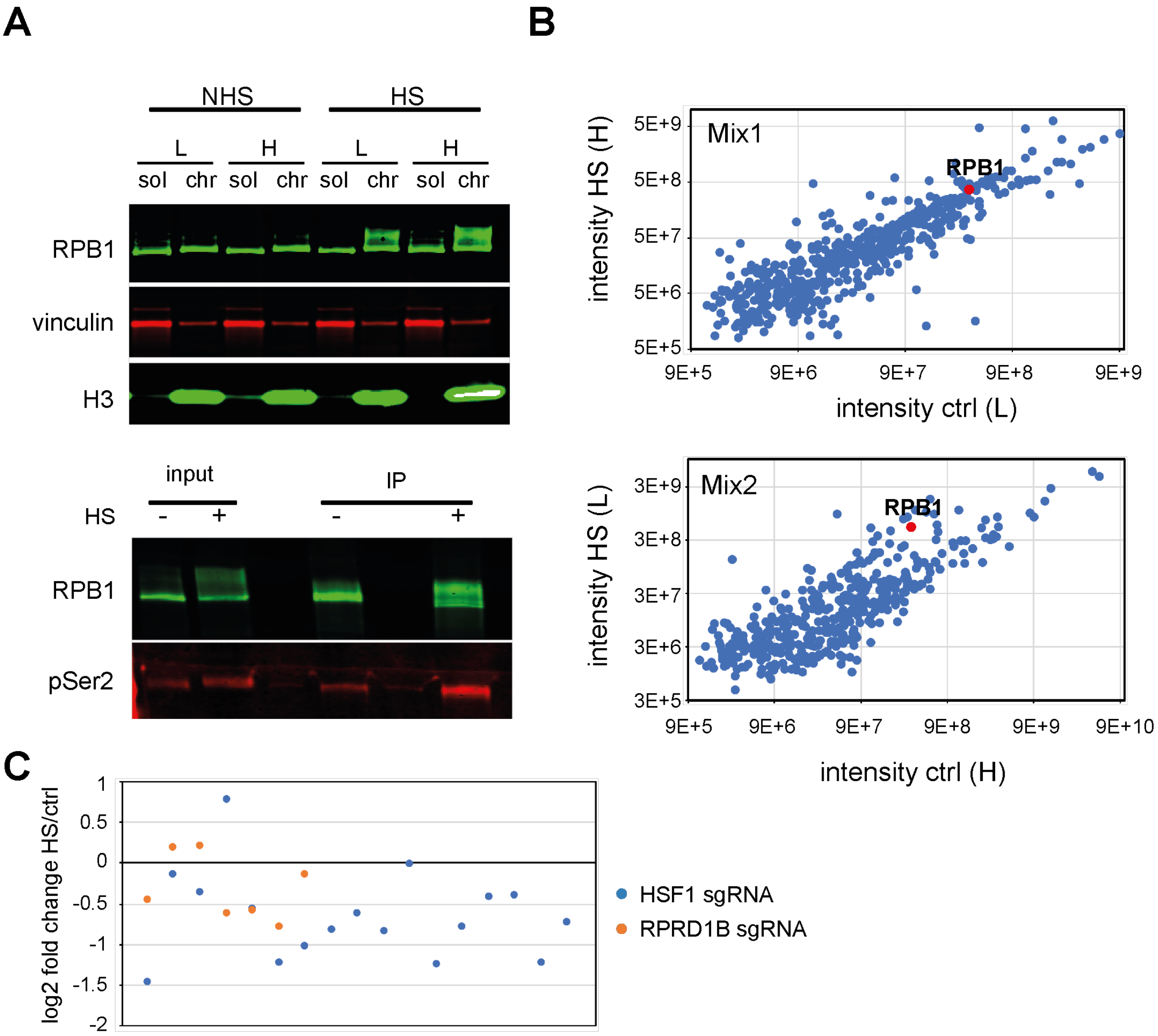

Figure S4. (*A,top*) Western blot analysis of chromatin fractionation used for IP-mass spec. Vinculin and H3 are loading controls. (*Bottom*) Western blot analysis of IP samples used for mass spec (*B*) Mass spec protein intensity distribution in the two mixes. RPB1 is highlighted by the red dot. (*C*) Relative abundance of single sgRNAs targeting HSF1 and RPRD1B. The experiments were carried out in MRC5-VA cells.

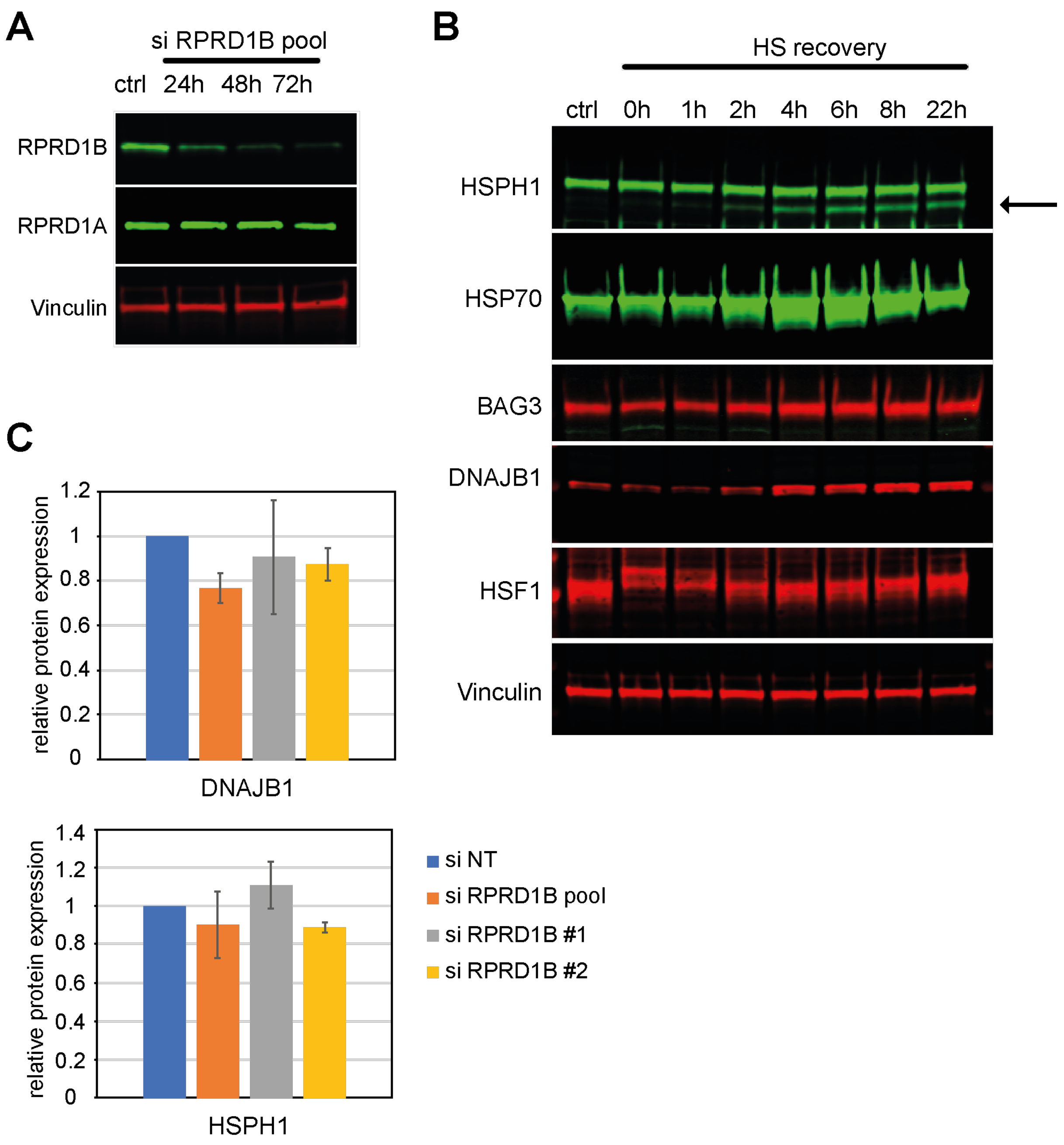

Figure S5. *(A)*Western blot analysis of RPRD1B depletion in whole cell extracts. (*B*) Western blot analysis of HSPs induction during a HS recovery time course in whole cell extracts. The arrow indicates the HS-induced isoform of HSPH1. Vinculin is used as a loading control. (*C*) Relative abundance of constitutive HSPH1and DNAJB1 in the absence of HS by quantification of fluorescence intensity. Average of three biological replicates. Error bars indicate ±SD. The experiments were carried out in MRC5-VA cells and siRPRD1B was depleted either with a mix of four siRNAs (pool) or two single siRNAs (#1 and #2).

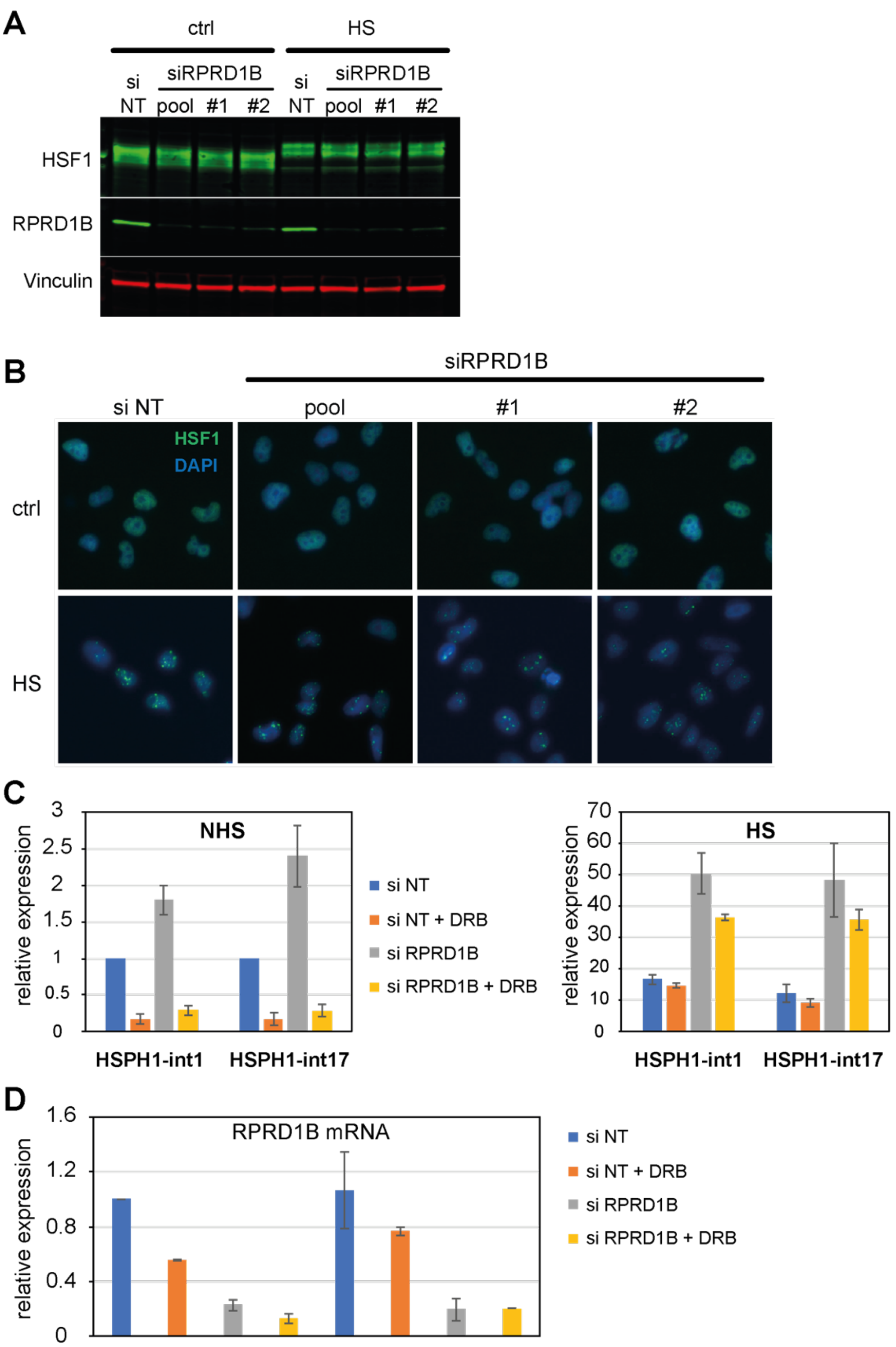

Figure S6. *(A)*Western blot analysis of HSF1 hyperphosphorylation shift after HS in whole cell extracts. Vinculin is used as a loading control. (*B*) Immunofluorescence analysis of HSF1 nuclear foci formation. *(C)* qPCR quantification of nascent RNA near the 5'-end or the 3'-end of the HSPH1 gene, relative to *GAPDH*, normalized to the control. *(D)* qPCR quantification of RPRD1B mRNA, relative to *GAPDH*, normalized to the control. Average of three biological replicates; error bars indicate ±SD. The experiments were carried out in MRC5-VA cells. For the western blot and the immunofluorescence analysis, siRPRD1B was depleted either with a mix of four siRNAs (pool) or two single siRNAs (#1 and #2). For the qPCR, siRPRD1B was depleted with a mix of siRNAs (#1 and #2).

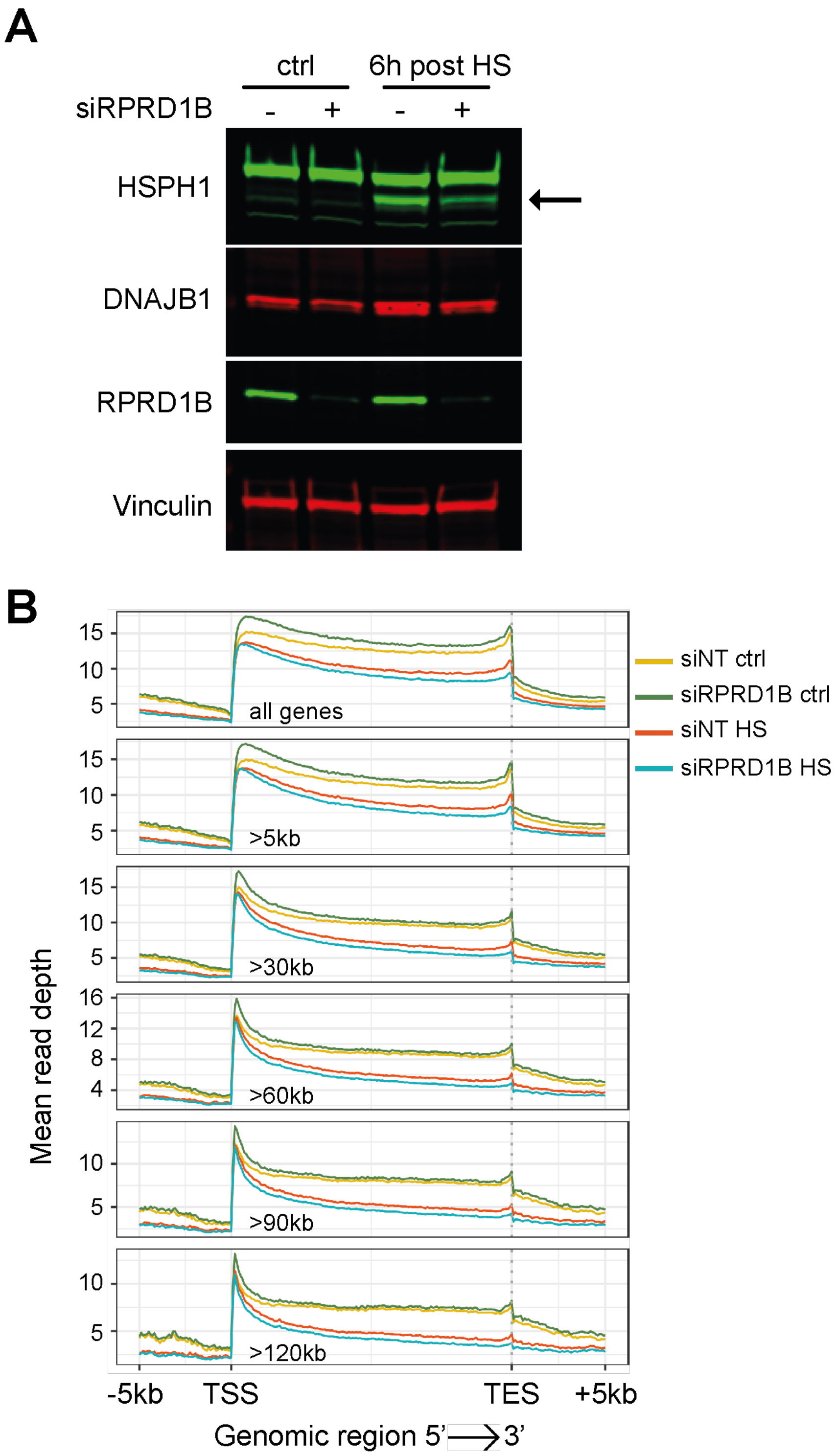

Figure S7. (*A*) Example of western blot analysis from cells used for TT_{chem}-seq and mRNA-seq analyses. The arrow indicates the HS-induced isoform of HSPH1. *(B)* Metagene profiles of TT_{chem}-seq stratified by gene length. The experiments were carried out in MRC5-VA cells and siRPRD1B was depleted with a mix of siRNAs (#1 and #2).

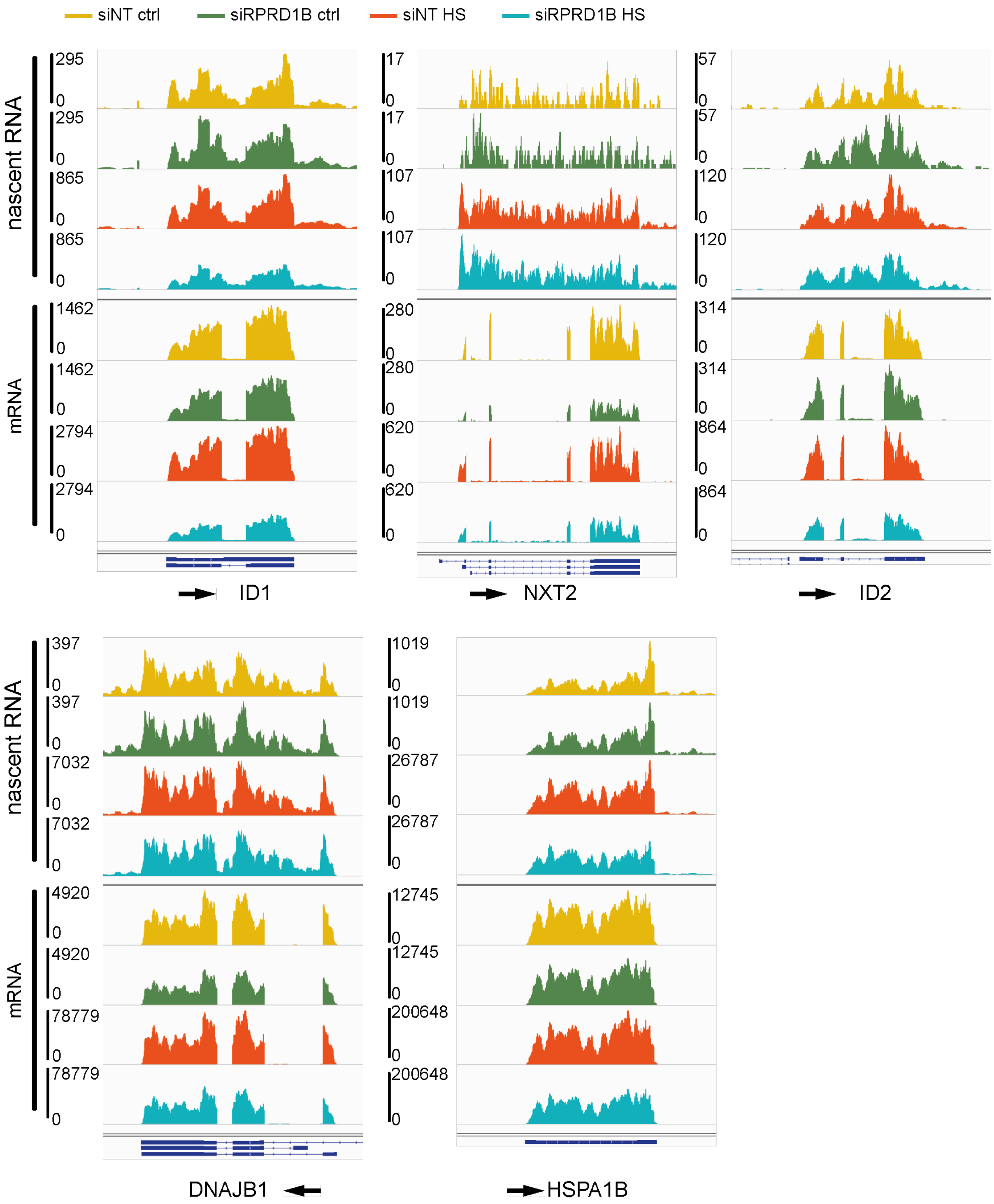

Figure S8. *(A)* IGV genome browser views of TT_{chem}-seq of the indicated genes. The experiments were carried out in MRC5-VA cells and siRPRD1B was depleted with a mix of siRNAs (#1 and #2).

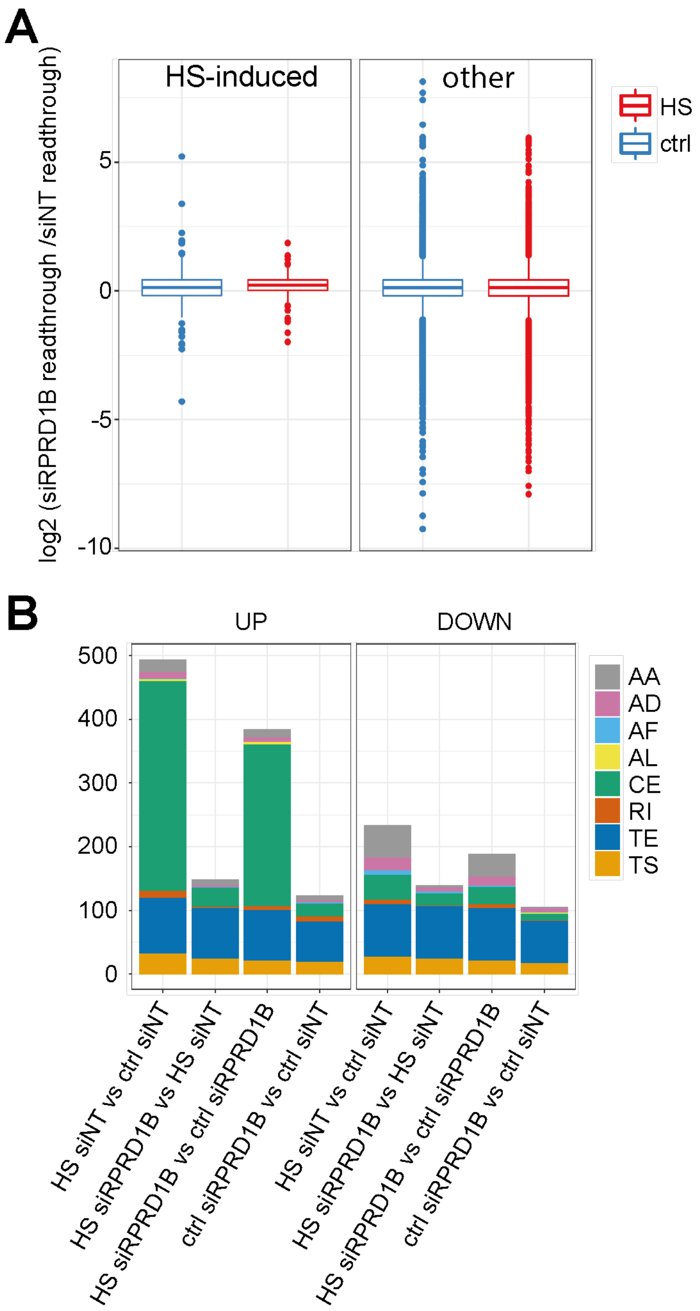

Figure S9. *(A)*Relative readthrough analysis for HS-induced and non-induced (other) genes using TT_{chem}-seq data. (*B*) mRNA isoform expression changes in the indicated samples by Whippet analysis of mRNA-seq data.

The experiments were carried out in MRC5-VA cells and siRPRD1B was depleted with a mix of siRNAs (#1 and #2).

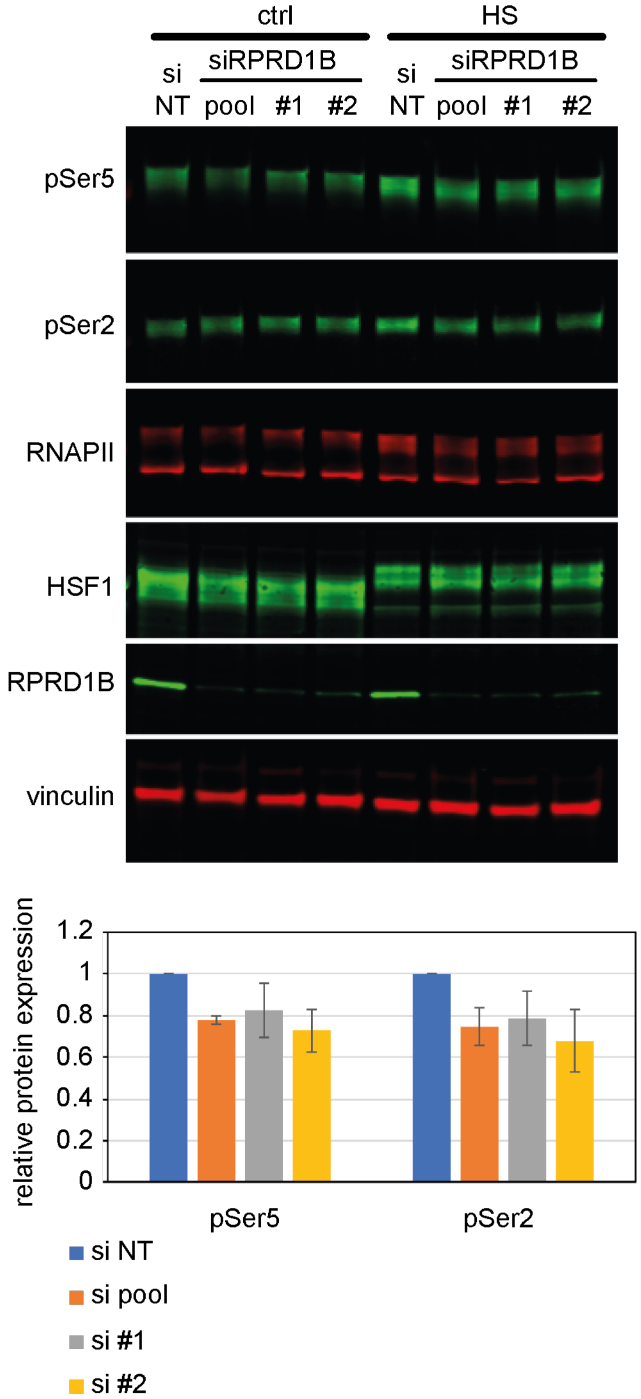

Figure S10. *(A, top)* Example of western blot analysis of RNAPII phosphorylation in whole cell extracts. Vinculin is used as a loading control. HSF1 and RPRD1B blots were used also in Figure S6. *(A, bottom)* Quantification of fluorescence intensity of the pSer5 and pSer2 markers normalized to vinculin, average of two biological replicates.

Supplemental Materials and Methods

RESOURCE AVAILABILITY

Lead Contact

Further information and requests for resources and reagents should be directed to and will be fulfilled by the Lead Contact, Jesper Svejstrup (jsvejstrup@sund.ku.dk)

Materials Availability

Cell lines generated in this study are available from the Lead Contact without restriction.

Data and Code Availability

Raw FASTQ files and unscaled bigwig files were deposited to the NCBI's Gene Expression Omnibus under accession number GSE197995.

EXPERIMENTAL MODEL AND SUBJECT DETAILS

Cell lines and culture conditions

HEK293T (human embryonic kidney epithelial, female origin), MRC5-VA (human fetal lung fibroblast, male origin), U2OS (human bone osteosarcoma epithelial, female origin) and their derivative cell lines were cultured in high glucose DMEM (Thermo fisher scientific) at 37°C with 5% CO_2. Culture media were supplemented with 10% v/v FBS, 100U/ml penicillin and 100mg/ml streptomycin. All cell lines were confirmed to be mycoplasma-free by the Francis Crick Institute Cell Services. For heat shock (HS), cells were subjected to immediate HS by replacing the media with pre-warmed media at 43°C and kept in an incubator at 43°C for the indicated period of time. For immunofluorescence experiments and clonogenic survival assays, cells seeded in 6-well plates were kept in their media and transferred into an incubator at 43°C for the indicated period of time. For the DRB treatment, cells were incubated in 100 µM DRB (Sigma-Aldrich, D1916) for 1,5 hours. The medium in the cells was then replaced either with medium at 37°C supplemented with 100 µM DRB in control samples or with medium pre-

warmed at 43ºC supplemented with 100 µM DRB in HS samples and the incubation was continued for additional 2 hours in an incubator at 37ºC or 43ºC. All the sequencing experiments were carried out in conditioned medium.

RNA interference

Generally, $8X10^5$ cells were transfected with siRNAs and harvested for different analyses 72 hours after transfection. 20 µM siRNA was mixed with Lipofectamine RNAiMAX (Thermo Fisher Scientific, 13778150) at a 1:3 (v/v) ratio according to the manufacturer's protocol, and the final concentration of siRNAs in the medium was 16 nM. The following siRNAs were used in the experiments: RPRD1B pool siRNA (Dharmacon, M-013787-01-0005), siRPRD1B single siRNAs (Dharmacon, D-013787-01-0020, D-013787-04-0020), control non-targeting siRNA (Dharmacon, D-001206-14-20).

Clonogenic survival assay

MRC5-VA cells transfected either with a pool of RPRD1B siRNAs or control siRNA were seeded at 200 cells/well into 6-well plates. Cells were exposed to HS for 2 hours and then kept in incubator at 37ºC for 11 to 13 days. Colonies were fixed by 4 % (v/v) formaldehyde and stained with a 0.1 % (w/v) crystal violet solution. Colonies from four biological replicates (each seeded into triplicate wells) were counted using a GelCount™ colony counter (Oxford Optronix Ltd) and normalised to untreated.

Western Blot

For whole cell extracts, cells were resuspended and incubated for 30 minutes at 4ºC in lysis buffer (250 U/ml BaseMuncher Benzonase (Expedeon, BM0100), 150 mM NaCl, 20mM TRIS-HCl pH 8, 0.1% (v/v) NP-40, 10% (v/v) Glycerol, 1.5 mM $MgCl_2$) supplemented with protease (Sigma-Aldrich, 05056489001) and phosphatase (PhosSTOP™, Sigma-Aldrich, 04906837001) inhibitors. The lysates were spun at 14000 RPM at 4ºC for 15 minutes and the supernatant was used for western blot analysis. Proteins were separated on 4%–15% TGX gels (BioRad, 5671084) and transferred either to a nitrocellulose membrane (GE Healthcare Life Sciences, 10600002) or to a PVDF membrane (Merck Life Science, IPFL00010). Membranes were blocked respectively in 5% (w/v) skimmed milk in PBS

supplemented with 0.1% (v/v) Tween20 (PBST) or in Intercept (PBS) blocking buffer (LI-COR, 927-70001) for 1 h at room temperature. Incubation with primary antibodies was carried out in PBST or Intercept (PBS) blocking buffer supplemented with 0.1% (v/v) Tween20 overnight at 4°C. Membranes were washed several times in PBST, incubated either with HRP-conjugated or LI-COR fluorescent dye-conjugated secondary antibodies diluted respectively in PBST or Intercept (PBS) blocking buffer supplemented with 0.1% (v/v) Tween20 for 45 min at room temperature and washed several times in PBST. Signal detection was obtained either using SuperSignal West Pico PLUS (Thermo Fsher Scientific, 34577) and Radiance plus (Azure Byosystems, AC2103) as ECL reagents, or Odyssey CLx imaging system (LI-COR). Primary antibodies used: HSF1 (Enzo Life Sciences, ADI-SPA-901); Vinculin (Sigma-Aldrich, V9131); RPRD1B (Bethyl, A303-782A-M); RPRD1A (Proteintech, 23652-1); Histone H3 (Abcam, ab1791); RPB1 N-terminal (Cell Signaling, D8L4Y); RPB1 Serine 2 phosphorylated, Serine 5 phosphorylated, Serine 7 phosphorylated and Tyrosine 1 phosphorylated are a kind gift from Dirk Eick; HSP70 (Proteintech, 25405-1); DNAJB1 (Proteintech, 67422-1); BAG3 (Proteintech, 10599-1); HSPH1 (Proteintech, 13383-1). Secondary antibodies used: anti-Mouse secondary antibody-HRP (Santa Cruz, sc-516102); anti-Rabbit secondary antibody-HRP (Jackson Immunoresearch, 711-035-152); anti-Rat secondary antibody-HRP (Jackson Immunoresearch, 112-035-003); IRDye 680RD Donkey anti-Mouse secondary antibody (LI-COR Biosciences, 926-68072); IRDye 800CW Goat anti-Rabbit secondary antibody (LI-COR Biosciences, 926-32211); IRDye 680RD Donkey anti-Rabbit secondary antibody (LI-COR Biosciences, 926-68073); IRDye 800CW Goat anti-Rat secondary antibody (LI-COR Biosciences, 926-32219); IRDye 680RD Goat anti-Rat secondary antibody (LI-COR Biosciences, 926-68076);

RT-qPCR

Total RNA was extracted using RNeasy mini kit (QIAGEN, 74106) following manufacturer instructions including an on-column DNase digestion (QIAGEN, 79254). 1 µg of RNA was used for Reverse transcription with TaqMan Reverse Transcription Reagents (Thermo Fisher Scientific, N8080234) according to manufacturer instructions. For detection of nascent RNA, random hexamers were used in the reverse transcription reaction and for the detection of mRNA, oligo dT primers. cDNA

was amplified on a CFX384 Touch Real-Time PCR Detector (BioRad 1855485) using iTAq Universal SYBR Green Supermix (BioRad, 172-5124) with the following conditions: 5 min denaturation at 95°C and 39 cycles of 10 s denaturation at 95°C, 10 s annealing at 58°C, and 20 s extensions at 72°C. Primers amplifying GAPDH mRNA were used for normalization purposes. All primer sequences are listed in Supplementary Table S7

TT_{chem}-seq

The TT_{chem}-seq was carried out as described in Gregersen et al., (2020) (Gregersen et al., 2020) in two biological replicates. MRC5-VA cells transfected either with a mix of two single RPRD1B siRNAs or control siRNA were seeded at density of 8 $X10^6$ /plate and were exposed to 2 hours of HS. The RNA was labelled *in vivo* with 1 mM 4SU (Glentham Life Sciences, GN6085) for 10 minutes prior to the addition of TRIzol (Thermo Fisher Scientific) that was used to stop the reaction at the desired time point. RNA was extracted according to manufacturer instructions.

As a control for equal sample preparation, we spiked-in 4-thiouracile (4TU) labelled RNA from *S. cerevisiae* (strain BY4741, MATa, his3D1, leu2D0, met15D0, ura3D0). The yeast culture was grown overnight in YPD medium, diluted to OD600 of 0.1 and grown to mid-log phase (OD600 of 0.8) and labelled 5 min with 5 mM 4TU (Sigma-Aldrich, 440736). Total RNA was extracted with PureLink RNA Mini kit (Thermo Fisher Scientific, 12183020) following the enzymatic protocol.

100 µg of human 4SU labelled RNA was spiked-in with 1 µg of 4TU-labelled yeast RNA and brought to a total volume of 100 µl with water. The mix was fragmented by adding 20 µl 1M NaOH and incubating in ice for 20 min. 80 µl of 1M Tris-HCl pH 6.8 were added to stop the fragmentation and samples were cleaned up twice with Micro Bio-Spin P30 Gel Columns (BioRad, 7326250) following manufacturer instructions. Biotinylation of 4SU and 4TU residues was carried out in a total volume of 250 µl 10 mM Tris-HCl pH 7.4 and 1 mM EDTA, containing MTSEA biotin-XXlinker (Biotium, BT90066) for 30 min at room temperature in the dark. The RNA was then purified by phenol:chloroform extraction, denatured 10 min at 65°C and added to 200 µl µMACS Streptavidine MicroBeads (Miltenyi Biotec, 130-074-101). After 15 min incubation at room temperature, the mix was loaded to a µColumn in the magnetic field of a µMACS magnetic separator. The beads were washed

twice in a buffer containing 100 mM Tris-HCl pH7.4, 10 mM EDTA, 1M NaCl and 01% Tween20. Biotinylated RNA was eluted twice in 100 mM DTT (200 µl final volume) and cleaned up with the RNeasy MinElute kit (QIAGEN, 74204) using 1050 µl 100% ethanol and 750 µl RLT buffer to precipitate RNA <200nt.

mRNA-seq

MRC5-VA cells transfected either with a mix of two single RPRD1B siRNAs or control siRNA were exposed to HS for 2h and then harvested by scraping in pre-warmed PBS and spun down. The pellets were then snap-frozen in liquid nitrogen and quickly defrosted for RNA extraction with RNeasy mini kit (QIAGEN, 79254) following manufacturer instructions including an on-column DNase digestion (QIAGEN, 79254). The RNA was used for library preparation.

Library preparation

For the TT_{chem}-seq experiment 30 to 100 ng (ask ASF) of 4SU/4TU labelled RNA were used for library preparation with the KAPA RNA HyperPrep kit (Roche, 08098107702) following manufacturer instructions with modifications as previously described (Tufegdzic Vidakovic et al., 2020). Briefly, the fragmentation step was omitted and the RNA, resuspended in FPE Buffer, was denatured at 65°C for 5 min. The two SPRI bead purifications were carried out, respectively, with a bead-to-sample volume ratio of 0.95x and 1x. For the mRNAseq experiment, 200ng of purified RNA per sample were used to prepare polyA+ mRNA libraries with KAPA mRNA HyperPrep kit (Roche, 08098123702) following manufacturer instructions. The libraries were then sequenced with single end 75bp reads on the Hiseq2500, with 50,000,000 reads per sample.

Chromatin fractionation

The cells were harvested by scraping in PBS, washed once with PBS and spun down. The pellets were then snap-frozen in liquid nitrogen. The quickly defrosted pellets were used to obtain a soluble fraction (containing cytosolic and nucleoplasmic proteins) and a chromatin fraction. Protease (Sigma-Aldrich, 05056489001) and phosphatase (PhosSTOP™,Sigma-Aldrich, 04906837001) inhibitors were added

fresh to all the buffers. The pellet volumes of buffer used refers to the original cell pellet volumes. 2 pellet volumes of hypotonic buffer were added (10 mM HEPES-KOH pH 7.5, 10 mM KCl, 1.5 mM $MgCl_2$) and the cells were incubated on ice for 15 min. To release cytosol, 20 strokes with a loose pestle were applied to the samples. To pellet nuclei, the samples were centrifuged at 3,900 rpm at 4°C for 15 min. The supernatant (cytosolic fraction) was collected, and pellets were resuspended by pipetting in 1.5 pellet volumes of nucleoplasmic extraction buffer (20 mM HEPES-KOH pH 7.5, 1.5 mM $MgCl_2$, 10% glycerol, 150 mM potassium acetate, 0.05% NP-40). 15 strokes with a loose pestle were applied to the samples, they were then incubated on ice for 20 min and cleared by centrifugation at 20,000g at 4°C for 20 min. The supernatant (nucleoplasmic fraction) was collected and combined with the cytoplasmic fraction after correcting this one to 10 % (v/v) glycerol, 3 mMEDTA, 0.05 % (v/v) NP-40 and 150 mM NaCl final concentration.

The chromatin pellets were resuspended in 1 pellet volume of chromatin digestion buffer [250 U/ml BaseMuncher Benzonase (Expedeon, BM0100) in 20 mM HEPES-KOH pH 7.5, 1.5 mM $MgCl_2$, 10% glycerol, 150 mM NaCl, 0.05% NP-40]. 15 strokes with a loose pestle were applied to the samples, which were then incubated on ice for 45 min and centrifuged at 20,000 g at 4°C for 20 min. The supernatant (low salt chromatin fraction) was collected and the remaining chromatin pellet was resuspended in 0.5 pellet volume of chromatin high salt chromatin extraction buffer (20 mM HEPES-KOH pH 7.5, 1.5 mM $MgCl_2$, 10% glycerol, 3 mM EDTA, 500 mM NaCl, 0.05% NP-40) and incubated on ice for 20 min. 2.3 pellet volumes of dilution buffer (20 mM HEPES-KOH pH 7.5, 1.5 mM $MgCl_2$, 10% glycerol, 3 mM EDTA, 0.05% NP-40) were added to the samples, and samples were then centrifuged at 20,000 g at 4°C for 15 min. Supernatant (high salt chromatin fraction) was collected and combined with the low salt chromatin fraction.

SILAC-based method for quantitative proteomic analysis

For mapping the RNAPII interactome in the presence of HS and DRB, MRC5-VA were cultured in SILAC light media or heavy media for 3 weeks. 97% efficiency of isotope incorporation was confirmed by mass spectrometry after 2 weeks in culture. The cells were then incubated with DRB either alone or in combination with HS before chromatin fractionation and the chromatin fraction was

used for the immunoprecipitation (IP). 1 mg of chromatin fraction was used per IP (all samples were adjusted to the same volume, typically 800-950 µl). For immunoprecipitation of RPB1 with 4H8 antibody, 100 µl of packed Protein G agarose beads (Thermo Fisher Scientific, 20397) per sample were prepared by washing twice in PBS supplemented with 0.05% Tween and then coupling to 30 µl of 4H8 antibody (1 mg/ml stock) for 1 h on a turning wheel at room temperature. The beads were then washed twice in PBS plus 0.05% Tween20 and once in IP buffer (20 mM Hepes-KOH pH 7.5, 1.5 mM $MgCl_2$, 10% glycerol, 150 mM NaCl, 0.05% NP-40) containing phosphatase and protease inhibitors. The beads were then resuspended in IP buffer containing phosphatase and protease inhibitors and added to the samples, to yield a 1 ml total reaction volume. The samples were incubated on a turning wheel in the cold room for 3 hours, and then centrifuged at 500 g at 4°C for 3 min. Supernatant (unbound fraction) was removed and saved, and the beads were washed 3 times in IP buffer. After the last wash, the beads were resuspended in 200 µl of IP buffer and loaded onto a Pierce™ Spin Columns-Screw cap (Thermo Fischer scientific, 69705) and spun 1 minute at 14000 rpm, the flowthrough was discarded. To elute the immunoprecipitated proteins, 50 µl of 2X Laemli buffer were added to the column, which were vortexed and boiled at 98°C for 5 min. The columns were centrifuged at 14000 rpm for 2 min and the elution was collected and used for western blot and mass spectrometry analysis. For mass spectrometry, light isotope-labeled samples and heavy isotope-labeled samples were mixed before loading on SDS-PAGE gel, for example, light labeled DRB-HS sample was mixed with heavy-labeled DRB sample; heavy-labeled DRB sample was mixed with light-labeled DRB-HS sample. The mixed samples were run around 10 mm into the fixed 10% NuPAGE Bis-Tris and stained with Instant Blue (Expedeon, ISB1L).

Mass spectrometry

Each lane of the submitted Coomassie-stained SDS-PAGE gel was excised into ten equal bands along its entire length using a scalpel. Twenty bands were transferred to separate 1.5 ml tubes and de-stained with successive washes of 100 mM aqueous ammonium bicarbonate (AMBIC) solution followed by ethanol solution. Proteins were simultaneously reduced and alkylated by incubating bands in aqueous 10 mM tris 2-carboxyethyl phosphine hydrochloride and 40 mM chloroacetamide solution on a

Thermomixer (70 ºC, 5 minutes, 1,000 rpm). Gel bands were washed by immersion in 50 % ethanol solution containing 50 mM AMBIC on a Thermomixer (22 ºC, 15 minutes, 1,000 rpm) then dehydrated by immersion in 100 % ethanol solution. Solutions were discarded after each incubation. A 20 µg vial of lyophilized trypsin (Pierce, MS Grade, 90057) was re-suspended in 50 mM acetic acid then diluted with 50 mM HEPES to produce a 2.5 ng/µl trypsin solution. 100 µl (250 ng) of trypsin was added to each gel band and the tubes were incubated on a Thermomixer (37 ºC, overnight, 1,000 rpm). The following morning, 50 µl of aqueous 25 % acetonitrile solution was added to each sample for peptide extraction. Tubes were placed in an ultrasonication bath for 5 minutes then the solution was transferred to new 1.5 ml tubes labelled "peptides". This extraction was repeated with a second 50 µl aliquot of aqueous 25 % acetonitrile solution and combined with the first. A final extraction using 50 µl of 100 % acetonitrile solution was performed. Peptide samples were dried via vacuum centrifugation then stored at -80 ºC.

Dried peptide samples were re-suspended in 200 µl of 0.1 % formic acid and loaded onto pre-equilibrated Evosep tips (Evosep, Denmark) using gentle centrifugation. An Evosep One robot loaded samples onto a C18 column (length 15 cm) and peptides were separated using the Evosep pre-defined 44-minute method. Eluted peptides were subsequently ionized and analyzed on a Q-Exactive orbitrap mass spectrometer (Thermo Scientific, USA) using a top 10 data-dependent acquisition method with settings: MS1 70k resolution, 1e6 AGC target, 250 millisecond maximum IT, 350-1800 m/z scan range, profile mode; MS2 35k resolution, 1e5 AGC target, 60 millisecond maximum IT, loop count 10, 200-2000 m/z scan range, NCE 33, profile mode. Forty.*raw* files were produced (one for each gel band).

Generation of Cas9-MRC5VA cell line

pCW57.1 dox inducible CAS9 vector and pLX-sgAAVS1 was purchased from Addgene. Cas9-MRC5VA cell lines were created by transduction of dox inducible flag-Cas9 vector. The cells were selected with Hygromycin for a week and plated at low density for clonal selection in a 15 cm dish. Several clones were recovered and tested for the expression of flag-Cas9 by western blot analysis. The clone with the strongest expression of flag-Cas9 was selected and further used in the screen.

Optimization of Cleavage efficiency by Cas9

For optimizing the cleavage efficiency by Cas9, virus expressing sg RNA targeting the AAVS1 locus was transduced to Cas9-MRC5VA cell line. The virus was transduced at 20% transduction efficiency. After 3 days of selection with blasticidin, Cas9 was induced with 1 μg/ml of doxycycline. Genomic DNA was extracted from cells after 1, 2-, 3-, 4- and 5-days post CAS9 induction. The AAVS1 locus was amplified using primers flanking the cleavage site (R-CCCCGTTCTCCTGTGGATTC, F-ATCCTCTCTGGCTCCATCGT). TIDE online software was used to detect the indels.

Virus production

pLX-sg-non targeting-A bunch of 30 non targeting plasmids was kindly provided by Paola Scaffidi. The Human CRISPR nuclear sub pool library was purchased from Addgene (Wang et al., 2014). Lentivirus of the nuclear sub pool was generated by co-transfection of the nuclear library sub pool with VSVG, GAG-POL and REV packaging plasmids into 293T cells using PIE (Polyethylenimine, Polysciences PIE-23966-1) transfection reagent. The media was refreshed once after 24 hours. 48 hours post transfection the viral supernatant was collected by passing it through 0.45 μm filters.

Pooled screening

The screen was performed in duplicates. 200 million target cells were transduced at 20% transduction efficiency with nuclear library viral pool in media containing 4μg/ml Polybrene (Sigma-Aldrich, H9268). After 3 days of selection with blasticidin, time zero (T0) cells were collected for genomic DNA extraction. Cas9 was induced with doxycycline (1μg/ml) for 5 days. CAS9 induced cells were split into two arms, untreated (UT) and heat shock (HS). The cells of the heat shock arm was incubated at 42°C for 4 hours. Cells were passaged every three days and after 10 days cells were harvested for genomic DNA extraction. To prepare the sgRNA libraries for NGS, a two-step nested PCR-based approach described by Wang, T et al was followed. Genomic DNA was isolated from 40 million cells. To ensure efficient amplification of the sgRNAs, 60 first PCR reactions were run for each sample using Phusion

DNA polymerase and a maximum of 1 µg gDNA in 50-µl reactions. Following the first PCR round, all reactions were pooled and 2 µl was used as template for the second PCR. Final products were pooled, run on a 2% agarose gel, excised, and purified using a QIAquick gel extraction kit (Qiagen, 28706) before sequencing. The libraries were analysed on a BioAnalyzer 2100 chip (Agilent) and then sequenced on an Illumina HiSeq 4000 platform using custom primers, generating ~30–50 million reads per sample.

Immunofluorescence staining

MRC5-VA cells were transfected with the indicated siRNAs according to siRNA interference protocol and then seeded on coverslips in a 6-well plate one day before harvesting. The cells were fixed with 3% paraformaldehyde in PBS for 10 minutes at room temperature. This was followed by permeabilization for 5 minutes with PBS supplemented with 0.5% Triton X-100. Cells were washed with 1 mL PBS for twice and blocked with PBS containing 5% milk for 30 minutes at room temperature. Primary antibodies (HSF1 Enzo Life Sciences Cat # ADI-SPA-901, 1:150) were diluted in PBS and incubated 30 minutes at room temperature. Cells were then washed twice in PBS supplemented with 0.1% Triton X-100 and incubated with secondary antibody in PBS (Donkey anti-Rabbit Alexa Fluor 488, Cat # A-21206 Thermo Fisher Scientific) for 30 minutes at room temperature. Cells were washed twice with 1 mL PBS supplemented with 0.1% Triton X-100 and the coverslips let air dry before mounting on glass slides with antifade mounting medium with DAPI (Vector Laboratories, H-1700). Images were acquired using micro-manager software (Edelstein et al., 2014) on a Zeiss Observer.Z1 wide-field microscope, equipped with a Plan-NEOFLUAR 20X/0.5 dry and a Plan-APOCHROMAT 40X/1.3 oil immersion objectives, selective bandpass filters for DAPI, GFP, RFP, Cy5, and a Hamamatsu Orca Spark CMOS camera. Images were visualized using FIJI open-source software (Schindelin et al., 2012).

QUANTIFICATION AND STATISTICAL ANALYSIS

TT-seq and mRNA-seq read alignment and quantification

Reads were processed using the publicly available nf-core rnseq pipeline v3.3 with the STAR/RSEM option against human genome assembly GRCh38 and Ensembl release 104 transcript annotations.

Other options were left as default expect for the TT-seq data which were simultaneously aligned against Saccharomyces cerevisiae R64-1-1 cdna sequences (Ensembl 104) using the "--additional_fasta" flag in order to quantify the yeast spike-in reads.

Data normalisation

The yeast spike spike-in was used to normalise sequencing depth between TT-seq samples. The RSEM estimated counts were read into R using the Bioconductor package tximport's "tximport" function, setting "type=rsem" but with otherwise default settings and converted to a DESeqDataSet object using DESeq2's "DESeqDataSetFromTximport" function. Genes with zero counts across all samples were dropped from the analysis and the object was further subset into yeast and human gene components. DESeq2's "estimateSizeFactors" function (default settings) was applied to the yeast object in order to calculate size-factors used to scale the read depth in the bigwig files (Love et al., 2014). The process was repeated for the human gene component, but the default size-factors generated by DESeq2 were replaced with the matched yeast scale-factor for the same sample before proceeding further. The process was repeated to generate scale-factors for the merged replicates by first creating a new counts matrix from the sum of the individual sample-level counts per replicate group. For the mRNA-seq samples the default DESeq2 size factors generated by counting against human genes were used.

BAM files merging

For the purposes of visualisation, genome alignment BAM files were merged across biological replicates, sorted and indexed using Picard v2.1.1 functions MergeSamFiles, SortSam and BuildBamIndex (all default settings) (http://broadinstitute.github.io/picard).

BigWig files

BigWig files were generated by converting BAM files to bedGraph format using BEDtools' genomecov function in a strand specific manner using the options "-bg –split -strand" (Quinlan and Hall, 2010). Where applicable, the reciprocals of the yeast scale factors were applied to normalize for differences in library size by additionally supplying them using the option "-scale". bedGraph files were in turn converted to bigWig format using the bedGraphToBigWig function (default settings) from the

KentTools package (Kent et al., 2010). All the genome-wide visualizations are strand-specific and represent a merge of at least two biological replicates.

DRB resistance analysis

Protein-coding genes were split into 2 groups based on their genomic width. "Short" genes were classified as those <20kb and a set of intervals were created to represent them running from their TSS to TES. "Long" genes >=20kb were similarly represented by a set of intervals stretching from the TSS to 20kb downstream. Median coverages over these intervals were calculated from the scaled bigwig files merged across replicate groups in a strand-specific fashion. More specifically, the scaled bigwig files were imported into R using the Bioconductor package rtracklayer's "import" function as an "RleList" and further manipulated using Granges to obtain median coverage over the defined intervals. A gene-wise ratio of the DRB/10' median coverages was then calculated separately for the HS condition. DRB-resistant genes were defined as those that showed a DRB/10' ratio of >= 0.35. a DRB median coverage of >1 and a 10' median coverage of >1 based on manual inspection, n=267.

Enrichment of the DRB resistant genes within the HS TT-seq data (Cugusi et al., 2022) was assessed using Gene Set Enrichment Analysis via the Bioconductor package fgsea (Korotkevich et al., 2019). The fgsea analysis involved ranking the Wald test statistic from the HS vs ctrl comparison ("stats" argument) and assessing where on that ranked list the DRB resistant genes ("pathway argument") mapped. Other fgsea options were left as default. DRB resistant genes showed a bias for being associated with genes induced under HS conditions, padj<1e-10 NES= 2.14.

mRNA-seq differential expression analysis

Gene-level RSEM estimated count data generated from the nf-core rnaseq pipeline were queried to identify protein-coding genes differentially expressed between replicate groups using the Bioconductor package DESeq2's Wald test. The RSEM estimated counts were read into R using the Bioconductor package tximport's "tximport" function, setting "type=rsem" but with otherwise default settings and converted to a DESeqDataSet object using DESeq2's "DESeqDataSetFromTximport" function. Genes with zero counts across all samples were dropped from the analysis. DESeq2's "estimateSizeFactors"

function (default settings) was object in order to calculate sample size-factors for normalisation. Significance was assessed based on a Wald test between replicate groups (design=0~group) using a FDR<0.05, together with a minimum mean abundance >= 100 and a fold-change of at least +/- 2. The log2FC values for the HS and NHS comparisons of siRPRD1B vs siNT groups were visualized in a gene-wise manner as a boxplot. Genes were stratified into those significantly "induced" due to HS in the control conditions (i.e. HS.siNT vs NHS.siNT) and "other". The significance of the difference between the gene-wise HS log2FCs and the NHS log2FCs for the "induced" and "other" groups was assessed using a Wilcoxon test, p<0.0001.

TT-seq differential expression analysis

TT-seq data were analysed as per the mRNA-seq data, except that a gene-level read count matrix was created directly from the BAM files using full genic intervals in a strand-specific manner. Scale-factors derived from the yeast spike-in genes were used to normalise the data prior to testing.

An alternative candidate set of DRB resistant genes were further refined by filtering for genes not significantly changing in the HS-10' vs HS-DRB comparison (FDR>0.05), but still well expressed across all 4 of the samples considered based on an FPKM filter (FPKM>=1). A heatmap of these 157 genes was generated from the gene-wise z-scores of the variance-stabilised counts across all samples. The variance stabilized counts were obtained from DESeq2's vst function with default settings. Genes appearing in the "previous" (see above) DRB resistance analysis were marked on the side of the heatmap.

Read depth profiles

Read depth profiles were created directly from the scaled bigwig files using deepTools v.2.5.3 (Ramirez et al., 2014) using gene definitions from Ensembl release 104 GTF. Metagene profiles were defined using the region 5kb upstream of the TSS, the gene-body divided into 100 equally sized bins and the 5kb downstream of the TSS (computeMatrix scale-regions -m 15000 -a 5000 -b 5000 --binSize 100 --

transcriptID gene --transcript_id_designator gene_id). Counts matrices for sense data were further subset for protein-coding genes greater than 5, 30, 60 and 90 kb in genomic width for visualisation.

Read-through analysis

The read-depth-ratio was defined as the ratio of read density of terminal exons divided by the read density in the downstream 20kb region. Only protein coding genes from standard chromosomes (1-22,X,Y) were considered. A single representative transcript was selected per gene, prioritising better transcript support level then larger transcript width. Coverage over these intervals were calculated from the scaled bigwig files merged across replicate groups in a strand-specific fashion. More specifically, the scaled bigwig files were imported into R using the Bioconductor package rtracklayer's "import" function as an "RleList" and further manipulated using Granges to obtain summed coverage over the defined intervals, then divided by each interval's width to obtain a coverage density. The read through ratio was then defined as the density in the terminal exon / density in the downstream region. Ratios of read-through ratios were used to compare between siRPRD1B and siNT samples for both HS and non-HS conditions. Data are presented for i) all genes and ii) 280 genes shown to be induced between HS/non-HS control conditions in the mRNA-seq analysis (FDR<0.05, log2FC>1 and baseMean>100).

Splicing analysis

Identification and quantification of splice-variants was conducted using the software Whippet v0.11.1 (Sterne-Weiler et al., 2018) running on Julia v0.6.4. A splice index was created using Gencode v39 basic gene annotation and the GRCh38 genome sequence using Whippet's whippet-index.jl function with default settings except for "--suppress-low-tsl". Each sample fastq file was quantified for PSI (percent spliced-in) values in turn using the whippet-quant.jl function with default settings except for "--biascorrect". Differential splicing events were assessed between replicate groups in a pairwise fashion by comparing PSI values using the "whippet-delta" function ("--min-reads 5, --min-samples 3, -g 123456"). Thresholding of the differential splicing results was conducted using the Bioconductor package GeneStructureTools (Signal, 2021), filtering the data based on a minimum probability >=0.99,

a change in PSI of >=0.3, a mean of at least 100 counts in one of the conditions to obtain a high confidence set.

sgRNA analysis

Raw reads from CRISPR libraries were trimmed to 20bp using cutadapt with the "--cut -<trim_size>" parameter. These were then mapped to the appropriate guide sequences using BWA (version 0.5.9-r16) (Li and Durbin, 2009) with the parameters "-l 20 -k 2 -n 2". sgRNA counts were obtained after filtering the mapped reads for those that had zero mismatches, and mapped to the reverse strand. The MAGeCK 'test' command (version 0.5.3) (Li et al., 2014) was used to perform the sgRNA ranking analysis between the relevant conditions with parameters "--norm-method total --remove-zero control".

CRISPR screen hit identification

sgRNAs depleted after gene KO were identified by comparing the normalized sgRNA counts in the Untreated (UT) population after CAS9 induction and Time zero (T0). Comparison of the UT with HS identified those sgRNAs which are depleted specifically in response to Heat shock treatment. Only sgRNAs with at least 10 raw counts in all the replicates at T_0 were used for analysis. Raw reads for each sgRNA were normalized to total read counts for each sample, and the fold change between UT and HS was calculated for each replicate. Depleted sgRNAs showing a $\log_2(FC) \leq -1$ in all the three triplicates were selected.

MASS SPECTROMETRY ANALYSIS

All *.raw* data files were analyzed using MaxQuant software version 1.6.0.3 (Ref 1). SILAC-specific quantification method settings were employed (multiplicity 2, Lys-8, Arg-10). Variable modifications of protein N-terminal acetylation and methionine oxidation, and fixed modification of cysteine carbamidomethylation were set. A SwissProt *Homo sapiens* protein database (downloaded January 2019) was queried using the default 1 % FDR at both protein and peptide levels. Results *.txt* files from MaxQuant were further analyzed in Perseus software

version 1.4.0.2 (Ref 2). Results were filtered to remove potential protein contaminants (including keratins), reversed sequences and “only-identified by site” hits. Protein intensities were log10 transformed and SILAC H/L ratios were log2 transformed.

References

Cugusi, S., Mitter, R., Kelly, G.P., Walker, J., Han, Z., Pisano, P., Wierer, M., Stewart, A., and Svejstrup, J.Q. (2022). Heat shock induces premature transcript termination and reconfigures the human transcriptome. Mol Cell.

Edelstein, A.D., Tsuchida, M.A., Amodaj, N., Pinkard, H., Vale, R.D., and Stuurman, N. (2014). Advanced methods of microscope control using muManager software. J Biol Methods *1*.

Gregersen, L.H., Mitter, R., and Svejstrup, J.Q. (2020). Using TTchem-seq for profiling nascent transcription and measuring transcript elongation. Nat Protoc *15*, 604-627.

Kent, W.J., Zweig, A.S., Barber, G., Hinrichs, A.S., and Karolchik, D. (2010). BigWig and BigBed: enabling browsing of large distributed datasets. Bioinformatics *26*, 2204-2207.

Korotkevich, G., Sukhov, V., and Sergushichev, A. (2019). Fast gene set enrichment analysis. bioRxiv, 060012.

Li, H., and Durbin, R. (2009). Fast and accurate short read alignment with Burrows-Wheeler transform. Bioinformatics *25*, 1754-1760.

Li, W., Xu, H., Xiao, T., Cong, L., Love, M.I., Zhang, F., Irizarry, R.A., Liu, J.S., Brown, M., and Liu, X.S. (2014). MAGeCK enables robust identification of essential genes from genome-scale CRISPR/Cas9 knockout screens. Genome Biol *15*, 554.

Love, M.I., Huber, W., and Anders, S. (2014). Moderated estimation of fold change and dispersion for RNA-seq data with DESeq2. Genome Biol *15*, 550.

Quinlan, A.R., and Hall, I.M. (2010). BEDTools: a flexible suite of utilities for comparing genomic features. Bioinformatics *26*, 841-842.

Ramirez, F., Dundar, F., Diehl, S., Gruning, B.A., and Manke, T. (2014). deepTools: a flexible platform for exploring deep-sequencing data. Nucleic Acids Res *42*, W187-191.

Schindelin, J., Arganda-Carreras, I., Frise, E., Kaynig, V., Longair, M., Pietzsch, T., Preibisch, S., Rueden, C., Saalfeld, S., Schmid, B.*, et al.* (2012). Fiji: an open-source platform for biological-image analysis. Nat Methods *9*, 676-682.

Signal, B. (2021). GeneStructureTools: Tools for spliced gene structure manipulation and analysis. R package version 1.14.0.

Sterne-Weiler, T., Weatheritt, R.J., Best, A.J., Ha, K.C.H., and Blencowe, B.J. (2018). Efficient and Accurate Quantitative Profiling of Alternative Splicing Patterns of Any Complexity on a Laptop. Mol Cell *72*, 187-200 e186.

Tufegdzic Vidakovic, A., Mitter, R., Kelly, G.P., Neumann, M., Harreman, M., Rodriguez-Martinez, M., Herlihy, A., Weems, J.C., Boeing, S., Encheva, V.*, et al.* (2020). Regulation of the RNAPII Pool Is Integral to the DNA Damage Response. Cell *180*, 1245-1261 e1221.

Wang, T., Wei, J.J., Sabatini, D.M., and Lander, E.S. (2014). Genetic screens in human cells using the CRISPR-Cas9 system. Science *343*, 80-84.
